# Time-dependent adaptations of damaged neurons and their microenvironment in the regenerating adult zebrafish spinal cord

**DOI:** 10.1126/sciadv.aea2882

**Published:** 2026-03-06

**Authors:** Leslie Lafouasse, Konstantinos Koutsogiannis, Yu-Wen E. Dai, Lisa Del Vecchio, Andrea Pedroni, Dimitrios Tsagkogiannis, Judith Habicher, Konstantinos Ampatzis

**Affiliations:** Department of Neuroscience, Karolinska Institutet, 171 77 Stockholm, Sweden.

## Abstract

Spinal cord injury (SCI) triggers complex cellular and extracellular responses that disrupt neuronal connectivity and hinder repair. While mammals have limited regenerative abilities, zebrafish achieve functional recovery through coordinated neuroprotection and plasticity. Here, we examined how structural and functional adaptations of damaged spinal neurons interact with extracellular matrix (ECM) dynamics during regeneration in adult zebrafish. We found that injured neurons undergo reversible changes in cellular properties and synaptic input, mediated mainly by glutamatergic signaling. These modifications coincide with a transient ECM reorganization marked by increased deposition of chondroitin sulfate proteoglycans (CSPGs). Enzymatic CSPG degradation paradoxically partially impaired long-term axonal regrowth and locomotor recovery. Thus, CSPG-rich ECM exerts a dual role: initially restricting plasticity but subsequently supporting structural stabilization and regeneration. Our findings highlight a temporally coordinated interplay between neuronal excitability, synaptic remodeling, and ECM reorganization as key determinants of spinal cord repair, offering mechanistic insights for enhancing nervous system regeneration.

## Teaser

Spinal cord healing in zebrafish depends on a perfectly timed interaction between neurons and their microenvironment.

## INTRODUCTION

Spinal cord injury (SCI) initiates a highly intricate and multifactorial cascade of events that develops over time, involving alterations at the intracellular, intercellular, and extracellular levels. These mechanisms collectively contribute to both the immediate effects of trauma and the subsequent long-term structural, biochemical, and physiological changes (*1*–*7*). Accordingly, the initial mechanical disruption, or primary injury, directly damages neurons, glia, and vascular structures. This insult is rapidly followed by a secondary phase of degeneration, during which a series of maladaptive cellular and molecular responses expands the lesion and amplify tissue loss in initially spared areas (*8*–*10*). Considerable research has focused on disentangling these processes to develop effective therapeutic interventions. However, despite substantial advances in identifying individual mechanisms of damage, translation into clinically effective treatments remains elusive, in large part due to the still limited understanding of how diverse injury-induced pathways dynamically interact in time and space (*3*, *5*, *11*). Among the many secondary injury mechanisms described, glutamate-mediated excitotoxicity has emerged as one of the most prominent and well-documented contributors to neuronal dysfunction and death (*10*–*16*). Yet, our current understanding remains incomplete regarding the detailed and intersecting mechanisms that operate at the lesion site, and there is insufficient clarity on how physically injured spinal neurons respond and adapt following injury.

Accordingly, the interplay between neuronal function and the extracellular matrix (ECM) has emerged as a central mechanism governing neuroplasticity under both physiological and pathophysiological conditions. The ECM is a mesh-like network of proteins, carbohydrates, and other macromolecules that not only provides structural support to tissues but also critically influences their mechanical properties (*17*–*19*). Far from being static, the ECM is highly dynamic and constantly remodeled to regulate essential cellular processes, including proliferation, migration, adhesion, morphogenesis, differentiation, and cell death (*17*, *20*). In the nervous system, specialized ECM aggregates envelop neuronal somata and proximal neurites, forming lattice-like structures (*19*). These structures are pivotal in stabilizing synaptic connectivity and modulating synaptic plasticity; they refine the timing and precision of neural signaling and act as protective barriers, preserving neuronal integrity through physical and electrochemical insulation (*19*, *21*–*24*). Following spinal injury, the ECM undergoes substantive alterations. In particular, there is a marked up-regulation of chondroitin sulfate proteoglycans (CSPGs), key molecules of the ECM, near the lesion site, creating a less permissive (inhibitory) extracellular milieu for synapse formation, axonal regrowth, and pathfinding (*1*, *21*, *25*–*28*). Despite this recognition, our understanding of the spatiotemporal dynamics of ECM remodeling and how this process interfaces with neuronal function remains limited, especially in species capable of innate regeneration (*29*, *30*).

Unlike mammals, zebrafish have a remarkable capacity to regenerate their spinal cord after injury (*6*, *31*–*35*). The complete recovery of the zebrafish spinal cord is not merely the result of extensive proliferation and neurogenesis (*31*, *36*, *37*); instead, it hinges on the dynamic structural and functional remodeling of spinal circuits (*6*). Critically, preserving neural integrity (neuroprotection) is central for enabling regeneration (*6*). It ensures that neurons remain viable, reorganize effectively, and allow newly generated neurons to be successfully integrated into existing networks. Thus, neuronal circuit plasticity forms the cornerstone of functional recovery in the regenerating spinal cord. This plasticity encompasses a diverse array of adaptive mechanisms, including intrinsic changes in neuronal excitability, synaptic reorganization, neurotransmitter switching, axonal regeneration, and the reestablishment of effective network connectivity (*6*, *30*, *33*, *35*, *38*–*41*). Such changes collectively contribute to and support the reconstitution of neural pathways, enabling the reemergence of coordinated motor functions and behaviors (*33*, *39*, *40*).

Here, we reasoned that a fundamental shift to a more holistic strategy is essential to address the existing challenges in SCI research. To this end, we used a multidisciplinary toolkit, combining anatomical, pharmacological, electrophysiological, and behavioral methods in adult zebrafish to map the temporal dynamics of spinal cord adaptive mechanisms and to understand how these mechanisms intersect and influence one another. This approach uncovered a complex, multilayered plasticity within the spinal neurons of adult zebrafish following injury, encompassing dynamic changes in neuronal activity, intrinsic excitability, synaptic remodeling, and reorganization of the ECM. Hence, our findings reveal an injury-induced cascade of temporally coordinated functional and structural adaptations that collectively drive the regenerative response. This integrative experimental approach, which captures the multifaceted nature of SCI pathophysiology, offers a strong framework for identifying synergistic molecular and cellular targets to support future therapeutic interventions aimed at improving spinal cord repair and functional recovery.

## RESULTS

**Fig. 1.**
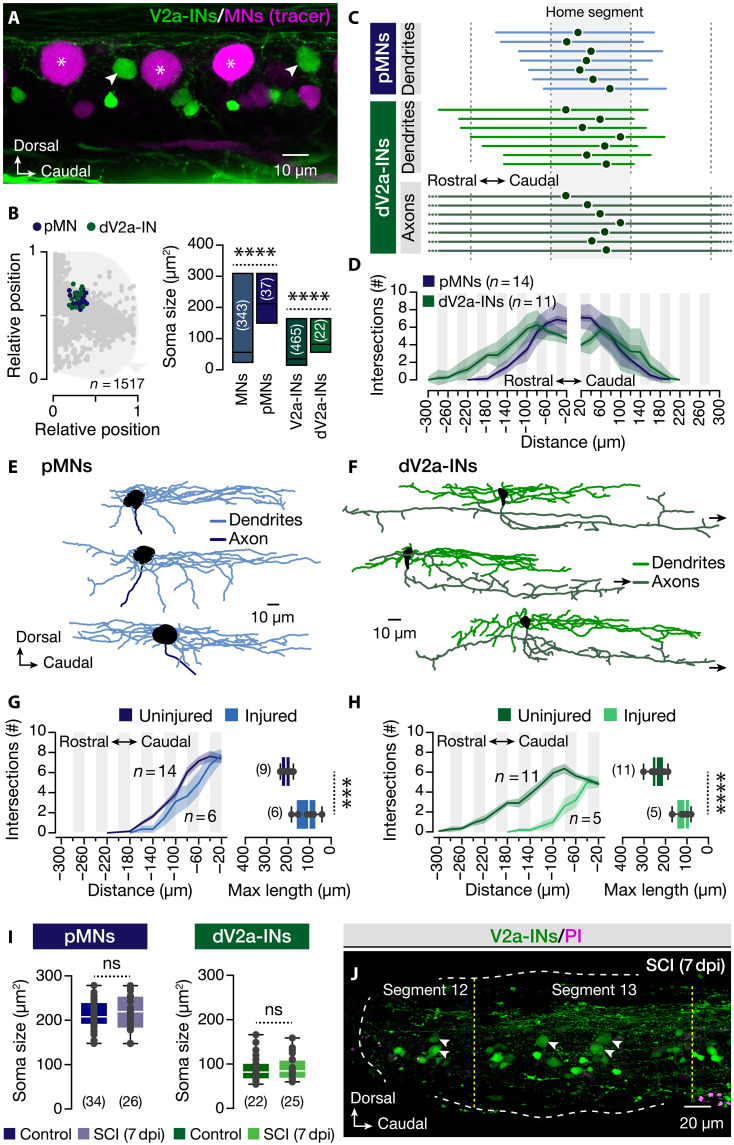
Anatomy of damaged spinal primary MNs and dorsal V2a-INs. (**A**) Whole-mount confocal image of adult zebrafish spinal cord showing V2a-INs and traced motoneurons. (**B**) Distribution patterns of the pMNs and dV2a-INs in the spinal cord, along with an analysis of their soma sizes in relation to other spinal neurons. (**C**) Plots display pMN and dV2a-IN soma (solid circle) with rostro-caudal dendrite and axon distribution. Lines show neurite position range. Dashed lines mark spinal cord segment borders. (**D**) Sholl analysis of the pMN and dV2a-IN rostro-caudal dendritic morphology. The light shaded area defines the SD, and the dark shaded area defines the SEM (solid line). (**E** and **F**) Morphological reconstructions of damaged pMNs and dV2a-INs close to the injured area. (**G** and **H**) Sholl analysis of the uninjured and damaged pMN and dV2a-IN dendritic morphology rostral to the neuronal soma. The light shaded area defines the SEM. Analysis of the maximum projection of dendrites located rostral to the neuronal soma. (**I**) Quantification of the pMN and dV2a-IN soma sizes in uninjured and 7 dpi animals. (**J**) Image of the adult zebrafish spinal cord shows the V2a-INs and the PI^+^ cells near the lesion site 7 dpi. Asterisks indicate the pMN cell bodies, and arrowheads indicate the dV2a-INs. GFP, green fluorescent protein; MN, motoneuron. Data are presented as floating bars (mean, minimum-maximum), and as box plots showing the median with 25/75 percentile (box and line) and minimum-maximum (whiskers). ***P < 0.001, and ****P < 0.0001. ns, not significant. For detailed statistics, see table S1.

In the adult zebrafish spinal cord, intact primary motoneurons (pMNs) exhibit dynamic, adaptive responses that safeguard their survival following injury (*6*). However, the cellular response of neurons that are physically damaged by traumatic SCI remains poorly characterized. To address this knowledge gap, we focused on and examined two spinal neuronal populations: the pMNs and the dorsally located V2a interneurons (dV2a-INs), which share a common distribution located in the dorsomedial motor column and have the largest somata among their respective classes (Fig. 1, A and B). Given their stereotypical morphology (Fig. 1, C and D), we hypothesized that a complete spinal cord transection at segment 12 would directly damage the rostrally projecting dendrites of both neuronal types, as well as the ascending axons of dV2a-INs situated near the lesion site (segments 12 to 13; ≤180 μm from the injury) (Fig. 1, C and D). To further confirm and evaluate the extent of neuronal damage, we reconstructed the morphology of neurobiotin-filled pMNs and dV2a-INs located caudal and adjacent to the lesion (Fig. 1, E and F, and fig. S1). Our analysis revealed an approximate 50% loss of rostral dendrites in both cell types and a clear disruption of the ascending axons in dV2a-INs (Fig. 1, G and H). Despite this neurite damage, soma size remained unchanged between uninjured and 7 days postinjury (dpi) neurons, indicating the preservation of the cell body integrity (Fig. 1I). Furthermore, consistent with prior evidence of pMN survival following injury (*6*), we observed no accumulation of the cell death marker propidium iodide (PI) in dV2a-INs, suggesting the absence of overt neuronal death (Fig. 1J). Collectively, these findings validate our injury paradigm as a model of partial, nonlethal neuronal damage, in which structurally compromised spinal neurons remain viable.

**Fig. 2.**
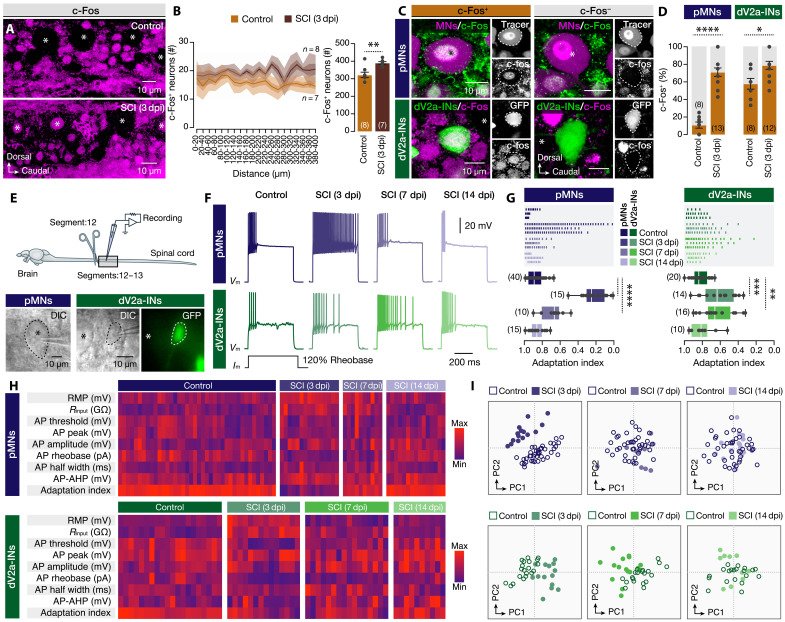
Changes in the damaged pMN and dV2a-IN activity and electrical properties after injury. (**A**) c-Fos immunoreactive spinal neurons in uninjured and 3 dpi animals. (**B**) Quantification of the number of c-Fos^+^ neurons caudally to the lesion site (excluding the damaged area of the lesion site) and comparison with the uninjured animals. The light shaded area defines the SD, and the dark shaded area defines the SEM (solid line). (**C**) High-magnification images show c-Fos^+^ and c-Fos^−^ immunolabeled nuclei. (**D**) Quantification of the proportion of c-Fos^+^ pMNs and dV2a-INs in uninjured and 3 dpi animals. (**E**) The experimental setup for the adult zebrafish spinal cord allows whole-cell patch-clamp recordings of putatively physically damaged pMNs and dV2a-INs near the injury site. The dashed line denotes the recorded neuron. Asterisk indicates the pMN. (**F**) Changes in the repetitive firing pattern occur in response to a depolarizing current step at 120% of the rheobase. (**G**) Representative sample of firing occurrences in three pMNs and dV2a-INs per experimental group and analysis of the adaptation index. (**H**) Normalized mean electrical properties for damaged pMNs and dV2a-INs across experiments. (**I**) PCA plots show clustering of damaged pMNs and dV2a-INs at 3, 7, and 14 dpi (color-coded solid circles) versus undamaged neurons (control, open circles). Asterisks mark pMN cell bodies. AHP, after hyperpolarization; AP, action potential; c-Fos, Fos proto-oncogene; DIC, differential interference contrast; PC, principal component; *R*input, input resistance. Data are presented as bar graphs (mean ± SEM), and box plots showing the median with 25/75 percentile (box and line) and minimum-maximum (whiskers). **P < 0.01, ***P < 0.001, and ****P < 0.0001. For detailed statistics, see table S1.

Next, we investigated potential changes in the activity of injured pMNs and dV2a-INs by first assessing the expression of c-Fos, a well-established surrogate marker of neuronal activation (*42*, *43*). Immunohistochemical analysis revealed a significant increase in the number of putative c-Fos^+^ spinal neurons caudal to the lesion site at 3 dpi (Fig. 2, A and B). We also observed an increase in the number of damaged pMNs and dV2a-INs expressing *c-Fos* at 3 dpi compared to uninjured controls (Fig. 2, C and D). Because of the fact that c-Fos expression serves as an indicator of indirect neuronal activity relating to transcriptional responses, we performed whole-cell patch-clamp electrophysiological recordings from the impaired pMNs and dV2a-INs to assess possible alterations in their excitability and electrical properties (Fig. 2E). In response to depolarizing current steps set at 120% of rheobase, both neuronal populations exhibited a marked increase in the number of action potentials at 3 and 7 dpi, accompanied by a significant reduction in firing adaptation (Fig. 2, F and G). By 14 dpi, the repetitive firing and adaptation dynamics had returned to baseline levels observed in uninjured animals (Fig. 2, F and G). Additional analyses of electrophysiological properties, including input resistance (*R*input) and rheobase, confirmed similarly dynamic alterations in both neuronal populations during the early postinjury phase (Fig. 2H and fig. S2). Notably, analysis of the resting membrane potential (RMP) showed a significantly more depolarized state in damaged neurons at 3 dpi, further indicating heightened neuronal excitability (Fig. 2H and fig. S2). To gain an integrated view of these changes, we performed principal components analysis (PCA) on nine electrophysiological parameters across all experimental conditions. PCA revealed a clear separation between injured (3 dpi) and control groups, with a progressive convergence of group profiles at 7 and 14 dpi, reflecting a temporal restoration of baseline electrophysiological states (Fig. 2I). Collectively, these findings demonstrate that SCI induces a dynamic yet reversible modulation of activity and excitability in damaged pMNs and dV2a-INs, suggesting that these early changes may play a critical role in the regenerative response.

**Fig. 3.**
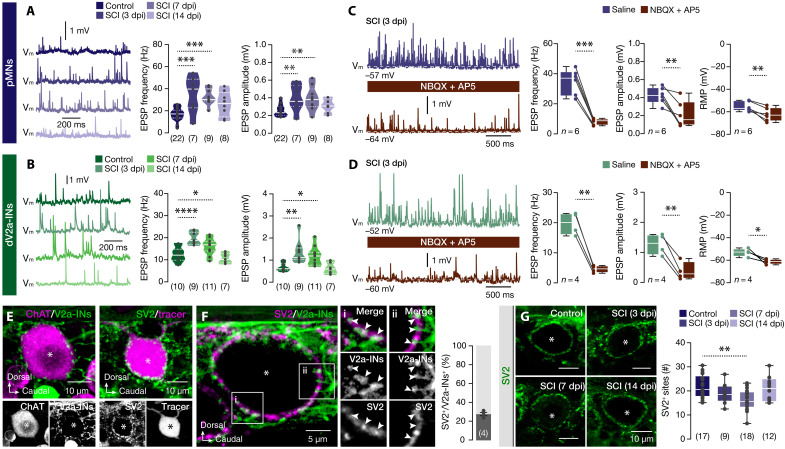
Synaptic and extrasynaptic changes in damaged pMNs and dV2a-INs. (**A** and **B**) Representative traces and analyses indicate changes in the frequency (in hertz) and amplitude (in millivolts) of recorded spontaneous EPSPs of the undamaged pMNs and dV2a-INs from uninjured (control) animals, as well as putative damaged pMNs and dV2a-INs after SCI. (**C** and **D**) Exogenous bath application of glutamate receptor antagonists NBQX and AP5, reduced the frequency (in hertz) and amplitude (in millivolts) of the recorded EPSPs in damaged pMNs and dV2a-INs at 3 dpi. Analysis shows changes in the RMP. (**E**) The immunofluorescent image displays the presence of V2a-IN (*chx10:GFP*^+^) axonal terminals (green) near the pMN (ChAT^+^, magenta) soma. Microphotograph showing the expression of SV2 (green) in retrogradely traced pMN soma (dextran, magenta). (**F**) Representative confocal image of the V2a-IN (*chx10:GFP*^+^) axonal terminals (green) and immunodetected SV2 (magenta) surrounding the pMN soma (asterisk) indicates that 27.5% of the SV2 presynaptic terminals are V2a-IN^+^ in proximity to the pMN soma. (**G**) Changes of the immunodetected SV2 expression in pMN somata (asterisks) under different experimental conditions. Analysis of the number of SV2^+^ aggregations in the pMN soma. ChAT, choline acetyltransferase; SV2, synaptic vesicle glycoprotein 2A. Asterisks indicate the pMN cell bodies. Data are presented as bar graphs (mean ± SEM), as violin plots, and as box plots showing the median with 25/75 percentiles (box and line) and minimum-maximum (whiskers). *P < 0.05, **P < 0.01, ***P < 0.001, and ****P < 0.0001. For detailed statistics, see table S1.

To determine whether the observed changes in activity and excitability of damaged pMNs and dV2a-INs were driven by altered synaptic input, we recorded the excitatory postsynaptic potentials (EPSPs) from both neuronal populations. At 3 and 7 dpi, both pMNs and dV2a-INs exhibited a significant increase in EPSP frequency and amplitude compared to uninjured controls, indicating enhanced excitatory synaptic drive (Fig. 3, A and B). By 14 dpi, both parameters had returned to baseline control levels (Fig. 3, A and B). To further characterize the nature of the excitatory inputs, we applied a pharmacological cocktail containing 2,3-dioxo-6-nitro-7-sulfamoyl-benzo [f]quinoxaline (NBQX) and D-(-)-2-amino-5-phosphonopentanoic acid (AP5), selective antagonists of AMPA/kainate and *N*-methyl-d-aspartate glutamate receptors, respectively. This intervention led to a ~75% reduction in EPSP frequency and a ~50% reduction in EPSP amplitude, confirming that most of the excitatory input was glutamatergic (Fig. 3, C and D). Notably, we also observed a significant hyperpolarization of the RMP following receptor blockade, suggesting a sustained, tonic glutamatergic extrasynaptic drive contributing to the depolarized state of the injured neurons (Fig. 3, C and D). Together, these results indicate that SCI triggers both synaptic and extrasynaptic glutamate release onto damaged pMNs and dV2a-INs, enhancing their excitability during the early phases of the regenerative response in the adult zebrafish spinal cord. This is consistent with previous reports showing that injury induces a surge of glutamate in mammals (*10*–*14*, *16*).

Next, we examined whether the increased excitatory input observed at 3 and 7 dpi in damaged pMNs and dV2a-INs was associated with structural changes in synaptic connectivity. To address this, we focused on pMNs, a well-characterized and readily accessible neuronal population that receives strong somatic excitatory input from V2a-INs, a glutamatergic interneuron class critical for pMN activation and recruitment during motor command execution (Fig. 3E) (*44*–*48*). Immunohistochemical labeling for synaptic vesicle protein 2 (SV2), a marker of presynaptic terminals, revealed a dense population of SV2^+^ puncta surrounding the pMN soma (Fig. 3E). Notably, 27.5% of these SV2^+^ terminals colocalized with green fluorescent protein (GFP)^+^ V2a-IN labeling, confirming the presence of somatic excitatory synapses (Fig. 3F). Quantitative analysis demonstrated a reduction in the number of perisomatic SV2^+^ puncta at 3 and 7 dpi, with values returning to baseline by 14 dpi (Fig. 3G). These findings suggest that the enhanced excitatory drive observed at early time points postinjury is not due to increased synapse number but is instead likely mediated by elevated glutamate release from existing presynaptic terminals.

**Fig. 4.**
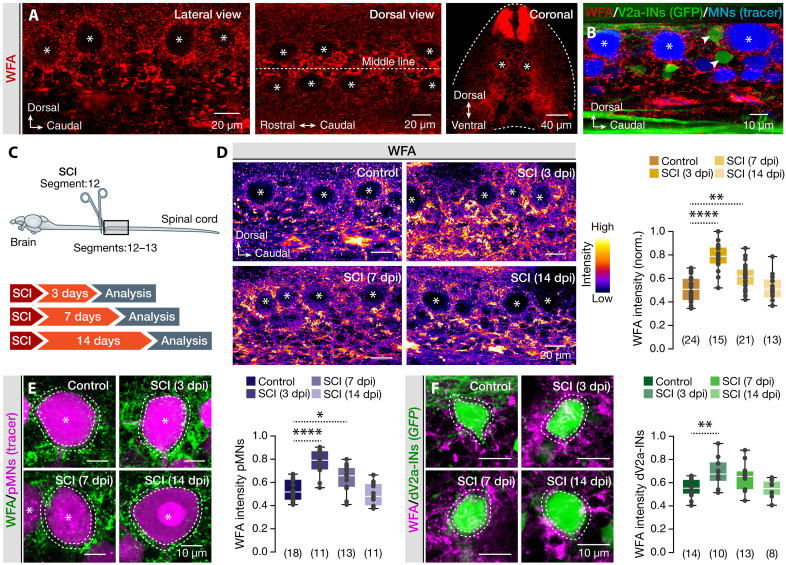
Postinjury ECM remodeling in the adult zebrafish spinal cord. (**A**) Representative confocal fluorescence images from whole-mount spinal cord and coronal sections show the WFA staining in the adult zebrafish spinal cord. (**B**) Fluorescent image created from a sample stack of the middle motor column area, showing the expression of WFA (red) near traced pMNs (blue, asterisks) and dV2a-INs (*chx10:GFP*^+^) (green, arrowheads). (**C**) Experimental approach to evaluate the changes in ECM through WFA lectin staining following SCI near the lesion site. (**D**) Representative pseudo-colored images illustrate the normalized intensity of WFA in the adult zebrafish spinal cord (at the level of the middle motor column) and an analysis of the WFA across all experimental conditions. (**E** and **F**) Changes in the detected ECM deposits (WFA^+^) around (2- to 3-μm perimeter; between the dotted lines) traced pMN and GFP^+^ dV2a-INs somata in different experimental conditions. Asterisks indicate the pMN cell bodies. Data are presented as box plots showing the median with 25/75 percentile (box and line) and minimum-maximum (whiskers). *P < 0.05, **P < 0.01, and ****P < 0.0001. For detailed statistics, see table S1.

Previous studies have established a causal link between ECM remodeling, synaptic formation, and plasticity, showing that the ECM not only regulates neuronal connectivity but also preserves neural integrity by acting as a physical and electrochemical barrier (*19*, *21*, *24*). Motivated by our findings on changes in the presynaptic terminals and the presence of extrasynaptic glutamate, which produced a tonic depolarization on damaged pMNs and dV2a-INs, we hypothesized that ECM remodeling could play a crucial role in modulating the observed injury-induced cellular and intercellular responses. To test this possibility, we used the plant lectin *Wisteria floribunda* agglutinin (WFA) to stain CSPGs, a major ECM component known to influence neural plasticity (*19*, *21*, *49*). WFA staining revealed a widespread distribution throughout the adult zebrafish spinal cord, with particularly high signal intensity in the midline and dorsal regions (Fig. 4A). Within the motor column, WFA was prominently detected in areas surrounding the somata of both pMNs and dV2a-INs, forming perineuronal-like (PNN-like) structures (Fig. 4B), as shown before in mammals (*19*, *22*, *23*, *50*, *51*). To assess ECM plasticity over time, we quantified normalized WFA intensity in the middle motor column at 3, 7, and 14 dpi (Fig. 4C). Our analysis revealed a dynamic and reversible remodeling of the ECM, characterized by a significant increase in WFA staining at 3 and 7 dpi, followed by a return to baseline levels by 14 dpi (Fig. 4D). Moreover, analysis of the perisomatic region (2- to 3-μm perimeter) of pMNs and dV2a-INs showed no correlation of the neuronal size with the WFA intensity (fig. S3A), and we observed a similar temporal pattern in damaged pMNs and dV2a-INs, with a peak in WFA intensity at 3 dpi and gradual normalization thereafter (Fig. 4, E and F). In addition to the overall increase in ECM deposition, we detected pronounced structural reorganization of the ECM/PNN-like structures, characterized by a shift from punctate patterns in uninjured tissue to elongated, filament-like formations after injury. This transformation likely reflects dynamic ECM remodeling that strengthens its role as a neuroprotective barrier, providing enhanced physical stabilization and biochemical insulation (fig. S3, B to D) (*22*, *51*–*56*). Together, these findings suggest that SCI induces a temporally coordinated remodeling of the ECM, which may serve to regulate both synaptic and extrasynaptic signaling. The concurrent changes in ECM structure, neuronal excitability, and synaptic input point to a potential causal relationship between extracellular, cellular, and intercellular mechanisms underlying the spinal cord’s regenerative response.

**Fig. 5.**
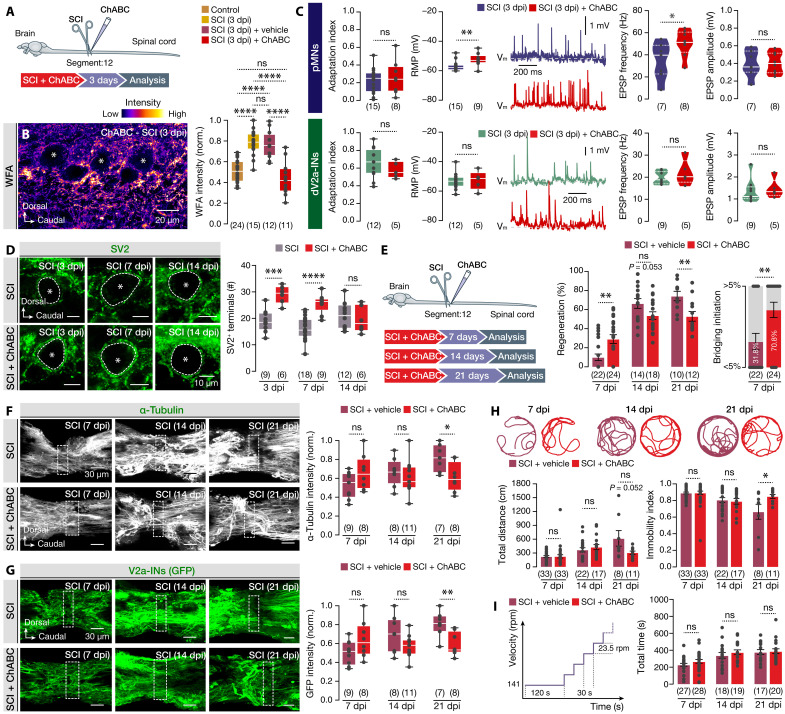
Physiological, anatomical, and behavioral changes following partial enzymatic digestion of ECM during spinal cord regeneration. (**A**) Experimental method for delivering ChABC at the lesion site. (**B**) Pseudo-colored WFA image and analysis show ECM partially degraded after 4 U of ChABC. (**C**) Changes in physiological properties and synaptic inputs after ChABC deglycosylation in pMNs and dV2a-INs at 3 dpi. (**D**) ChABC digestion rescues the reduction of the SV2^+^ terminals of the pMNs at 3 and 7 dpi. (**E**) Experimental approach for enzymatic digestion of ECM on spinal cord bridge formation [regeneration index (RI)] at different time points after injury. At 7 dpi, ChABC treatment facilitates the initiation of the bridge formation. (**F** and **G**) Whole-mount microphotographs of the zebrafish spinal cord show the α-tubulin^+^ structures and the V2a-IN^+^ neurites (GFP^+^) crossing the lesion site (dotted box) and their intensity during spinal cord regeneration. (**H**) Representative traces of injured zebrafish swimming during the open-field test and analysis of total distance traveled and the immobility index at different time points after injury between vehicle control and ChABC-treated animals. (**I**) Modified swim tunnel test to assess the forced swimming performance of injured zebrafish during spinal cord regeneration in vehicle-treated and ChABC-treated animals. Asterisks indicate the pMN cell bodies. Data are presented as bar graphs (mean ± SEM), as violin plots, and as box plots showing the median with 25/75 percentiles (box and line) and minimum-maximum (whiskers). *P < 0.05, **P < 0.01, ***P < 0.001, and ****P < 0.0001. For detailed statistics, see table S1.

To determine whether ECM remodeling causally contributes to the altered excitability and synaptic input of damaged pMNs and dV2a-INs, we developed a targeted approach to partially degrade CSPGs using the bacterial enzyme chondroitinase ABC (ChABC) from *Proteus vulgaris* (*57*, *58*). Intraspinal administration of ChABC at the lesion site, but not of the control vehicle, led to a dose- and spatially dependent reduction in WFA intensity within the motor column (Fig. 5, A and B, and fig. S4). Notably, treatment with 4 U of ChABC restored ECM levels to those observed in uninjured control animals in the evaluated area of the adult zebrafish spinal cord (Fig. 5B). To assess the impact of perineuronal ECM up-regulation in the spinal cord postinjury, we recorded damaged pMNs and dV2a-INs after injury (3 dpi) in ChABC-treated animals. Although no significant changes in firing adaptability were observed in either population, we noted a substantial depolarization of the RMP in pMNs (Fig. 5C) that are characterized by dense ECM depositions surrounding their somata (fig. S3, B to D), but not in the control vehicle–treated animals (fig. S5). Further analysis of excitatory synaptic events revealed a significant increase in EPSP frequency in pMNs following ChABC treatment, indicating that partial ECM digestion enhances excitatory synaptic input (Fig. 5C). To probe whether perineuronal ECM degradation also affects structural synaptic remodeling, we quantified SV2^+^ presynaptic terminals around the somata of damaged pMNs. Partial deglycosylation following ChABC treatment effectively rescued the injury-induced reduction in SV2^+^ contacts observed at 3 and 7 dpi (Fig. 5D). Together, these results highlight a pivotal role for ECM remodeling in modulating both the electrophysiological properties and synaptic plasticity of damaged spinal neurons. Specifically, CSPG-rich ECM regulates tonic depolarization, excitatory input, and presynaptic structural remodeling, particularly in pMNs, during the early phases of regeneration.

In the mammalian spinal cord, excessive deposition of CSPGs at the lesion site is widely recognized as a major inhibitory factor that impedes axonal regeneration (*59*–*61*). However, how this ECM response influences regeneration in species with inherent regenerative capacity, such as the adult zebrafish (*6*, *31*–*34*), remains poorly understood. To address this, we first assessed the impact of ECM digestion on regenerative progression by quantifying spinal cord bridging as a proxy for tissue repair. ChABC-treated animals exhibited a significant acceleration in bridging at 7 dpi, suggesting an early facilitation of regeneration (Fig. 5E). Unexpectedly, this initial phase was succeeded by a notable stagnation in regeneration at later stages (Fig. 5E). By 21 dpi, ChABC-treated animals showed a significant delay in spinal cord bridging compared to control vehicle, which continued to regenerate without interruption (Fig. 5E). To investigate whether this delayed regeneration was associated with impaired axonal regrowth, we quantified the density of neurites crossing the lesion site using α-tubulin immunostaining and GFP labeling of V2a-INs. Consistent with the early bridging phenotype, ChABC-treated animals displayed an initial increasing trend in neurite crossing at 7 dpi (Fig. 5, F and G). However, this was followed by a marked reduction in regrowth progression at 14 and 21 dpi, in contrast to the continuous increase observed in untreated animals (Fig. 5, F and G). To evaluate the functional relevance of these anatomical differences, we conducted open-field and forced swim tests to assess locomotor behavior and recovery. Both experimental groups (control/vehicle treated and ChABC treated) exhibited progressive improvements in most swimming parameters over time (fig. S6). Yet, by 21 dpi, ChABC-treated animals showed a marked reduction in total distance traveled (*P* = 0.052; for detailed statistics, see table S1) and increased immobility compared to the controls (Fig. 5H). In contrast, forced swim performance was indistinguishable between groups (Fig. 5I), suggesting context-dependent functional consequences of ECM modulation. Together, these findings support a differential role for CSPG-rich ECM in spinal cord regeneration, with the early removal of ECM transiently enhancing the onset of regeneration; however, sustained ECM integrity appears essential for continued axonal crossing and functional recovery. These results argue against indiscriminate ECM degradation as a therapeutic strategy for central nervous system repair and underscore the importance of temporally tuned ECM remodeling for successful regeneration of the spinal cord.

## DISCUSSION

We have identified a complex and multilayered plasticity in neuronal activity, excitability, synaptic remodeling, and ECM reorganization within spinal neurons following injury in adult zebrafish, a vertebrate model known for its innate regenerative capabilities (*6*, *31*–*35*). By focusing on two critical neuronal populations—the pMNs and the dV2a-INs—we demonstrate an injury-induced cascade of functional and structural adaptations that occur in a temporally coordinated manner during the regenerative process. Our findings offer further insights and expand the mechanistic framework established previously, which highlighted a gap junction–mediated neuroprotective mechanism among undamaged spinal neurons adjacent to the lesion site in adult zebrafish (*6*).

In line with our previous findings (*6*), damaged neurons exhibited a transient surge in glutamatergic input and heightened excitability without showing signs of morphological degeneration or cell death. Notably, the electrophysiological properties of the undamaged pMNs returned to baseline levels by 7 dpi (*6*). In contrast, neurons that sustained direct damage in the present study required an extended recovery period, with normalization of excitability and synaptic properties observed by 14 dpi. These temporally distinct recovery trajectories highlight a phased and coordinated remodeling process: undamaged zebrafish spinal neurons rapidly stabilize local circuit activity, exerting a neuroprotective buffering effect that limits the spread of potential degeneration (*6*). This early, circuit-level neuroprotective strategy represents a regenerative mechanism that is distinctly different from the processes occurring in the mammalian spinal cord during the secondary injury phase (*8*–*10*). Meanwhile, damaged neurons appear to engage in delayed but robust structural and functional regeneration. Collectively, our current and prior findings (*6*) underscore the essential interplay between neuronal resilience, neuroprotection, and circuit plasticity as a cornerstone of successful spinal cord regeneration.

In addition to the postinjury enhancement of intrinsic excitability in damaged neurons observed at 3 and 7 dpi, we also detected an early increase in c-Fos expression, underscoring an immediate response that encompasses not only electrophysiological changes but also transcriptional activity, suggesting a heightened state of neuronal activation. Beyond serving as a classical marker of neuronal activity (*42*, *43*), c-Fos has also been linked to neuroprotection and neuronal repair. Previous studies have shown that c-Fos is crucial for regulating DNA damage repair and neuronal survival and that its overexpression confers protective effects in vulnerable neural populations (*62*, *63*). Consequently, the observed increase of c-Fos^+^ neurons in our model likely reflects more than just enhanced neuronal excitability; it may signify a dual neuroprotective response that initiates transcriptional programs critical for cellular repair while supporting the survival of functionally active neurons during the early regenerative phase, a period characterized by elevated metabolic demand and excitotoxic vulnerability (*10*, *11*, *13*, *14*). We also found an inverse relationship between dynamic synaptic excitatory input, primarily mediated by glutamatergic transmission, and a decrease in SV2^+^ presynaptic contacts. This indicates that the increased excitatory drive stems from enhanced neurotransmitter release at existing synapses, potentially through compensatory mechanisms such as presynaptic facilitation or altered vesicle dynamics (*64*–*66*). Simultaneously, we noted tonic depolarization indicative of extrasynaptic glutamatergic signaling. Accordingly, in pathological conditions such as traumatic injury, elevated levels of glutamate are released into the spinal cord parenchyma from various cellular sources, including damaged neurons, astrocytes, reactive microglia, cerebrospinal fluid, and through proteolytic mechanisms (*11*, *12*, *67*–*71*). This aberrant release causes a significant increase in extracellular glutamate, which is a characteristic feature leading to disrupted neural homeostasis and excitotoxicity in mammals (*11*, *12*, *67*, *68*, *72*). However, the precise cellular sources and mechanisms underlying this form of volume transmission in zebrafish remain to be clarified.

Our data provide essential insights into the nuanced role of the ECM in modulating neuronal excitability and synaptic plasticity after SCI, refining the current understanding of both the therapeutic potential and inherent limitations of ECM-targeted interventions. We observed that the accumulation of CSPGs temporally coincided with heightened excitability and synaptic reorganization. The temporally shifted interaction between pMNs’ ECM remodeling and presynaptic structural changes (Figs. 3G and 4E) suggests that perineuronal ECM remodeling happens earlier and likely directs synaptic plasticity, rather than occurring alongside it as an independent process. This supports a causal role for ECM dynamics in guiding synapse formation, stabilizing synaptic connectivity, and fine-tuning neuronal signaling, as previously shown (*19*, *23*, *24*, *51*, *73*). In line with these observations, we found that partial enzymatic digestion of the ECM using ChABC selectively modulated the frequency of excitatory synaptic inputs and rescued the presynaptic terminal density in pMNs, demonstrating a causal link between ECM reorganization and synaptic architecture. Furthermore, ECM deglycosylation led to a significant depolarization of the RMP in pMNs, a neuronal subtype marked by dense perineuronal ECM, supporting the view that the ECM may act as a neuroprotective barrier by providing both physical and biochemical insulation, consistent with earlier findings (*22*, *51*–*56*). Despite the apparent effects of SCI on decreasing firing adaptability in pMNs at 3 dpi, our data also indicate that enzymatic degradation of the perineuronal ECM structures with ChABC does not restore it. This suggests that firing adaptability is not primarily governed by the structural integrity of ECM components but is more likely influenced by SCI-induced changes in ion channel conductances and their dynamics, which ultimately determine how neurons modify their firing patterns over time.

Questioning the importance of ECM remodeling in spinal cord regeneration, we found that ChABC-treated injured animals displayed a transient facilitation of the regeneration process by accelerating bridge formation and neurite outgrowth, as shown before (*74*–*78*). However, our findings also revealed that long-term regeneration and functional recovery were partially impaired, suggesting that although injury triggers a cascade of conserved cellular responses across vertebrates, using a regenerative model, such as the adult zebrafish, offers unique insights into the long-term consequences of ECM manipulation. Hence, our findings demonstrate a paradoxical dual functional role of the ECM within the context of the spinal cord. This is mediated by a rapid, injury-induced up-regulation of CSPGs during the early phase, as reflected by the increased WFA labeling, which likely stabilizes the extracellular microenvironment and protects neurons, mitigating potential excitotoxic damage. As recovery progresses, the gradual normalization of CSPG levels indicates an active remodeling process that supports synaptic plasticity, axonal and dendritic regrowth, and the integration of newly generated neurons. Thus, the temporal dynamics of ECM remodeling reflect a finely tuned balance between neuroprotection in the acute phase and facilitation of regeneration during later stages. Collectively, these results underscore the potential hazards linked to excessive or indiscriminate degradation of the ECM, which could inadvertently enhance maladaptive plasticity and increase neuronal susceptibility. While ECM manipulation presents a promising therapeutic strategy, it is critical to implement temporally regulated, cell type–specific approaches that retain the ECM’s protective characteristics while unlocking its regenerative capacity. Future therapeutic interventions should aim to establish this intricate balance to optimize functional recovery without compromising the structural and physiological integrity of neural circuits.

An essential and unresolved inquiry pertains to the cellular origins of ECM components that undergo dynamic remodeling in response to SCI. Although astrocytes were initially recognized as the primary producers of proteoglycans (*25*, *79*, *80*), studies suggest that macrophages, oligodendrocyte progenitor cells, and microglia also serve as substantial contributors to the production of these proteoglycans (*81*, *82*). These findings highlight the complex regulation of ECM composition specific to different cell types. Considering the substantial remodeling of the perineuronal ECM surrounding damaged spinal neurons in our model, and its potential as a therapeutic target, it is crucial to differentiate the contributions of individual cell types. This distinction is key to understanding the cellular-extracellular interactions that affect spinal cord repair.

## MATERIALS AND METHODS

### Animals

Adult wild-type zebrafish (*Danio rerio*; AB/Tübingen strain, RRID: ZIRC_ZL1), along with transgenic Tg (*chx10:GFP^nns1^*, ID: ZDB-ALT-061204-2) zebrafish, aged 8 to 12 weeks (total length: 13 to 20 mm) of both sexes, were used in this study. All animals were size-matched within each experimental group to minimize variability due to age or body size. No additional inclusion criteria or randomization/blinding procedures were applied during group assignment. All experimental protocols were approved by the Swedish Regional Ethical Committee in Stockholm (permit numbers: 9248-2017, 19535-2020, and 7650-2022) and were conducted in accordance with the European Directive 2010/63/EU on the protection of animals used for scientific purposes, as well as the Animal Research: Reporting of In Vivo Experiments (ARRIVE) guidelines. The number of animals per experimental procedure was carefully determined to balance ethical considerations with scientific rigor. Sample sizes were calculated on the basis of preliminary data and prior studies to ensure robust statistical power while minimizing animal use.

### Motoneuron retrograde tracing

Retrograde labeling of spinal motoneurons was conducted in anesthetized zebrafish using 0.03% tricaine methane sulfonate (MS-222; E10521, Sigma-Aldrich). Tracers, either tetramethylrhodamine-dextran (molecular weight, 3000; D3307, Thermo Fisher Scientific) or biotinylated dextran (molecular weight, 3000; D7135, Thermo Fisher Scientific), were injected directly into the axial muscles or ventral roots corresponding to the relevant myotomes. Following injection, animals were maintained for 4 to 6 hours to permit retrograde transport of the dye to the motoneuron somata. For biotinylated dextran-labeled tissue, spinal cords were rinsed extensively in phosphate-buffered saline [PBS; 0.01 M (pH 7.4); sc24946, Santa Cruz Biotechnology Inc.) and subsequently fixed in 4% paraformaldehyde (PFA; CAS30525-89-4, Santa Cruz Biotechnology Inc.) in PBS overnight at 4°C. Fixed tissues were then incubated with Alexa Fluor–conjugated streptavidin (1:500; see table S2) overnight at 4°C in the dark to visualize the tracer.

### Spinal cord injury

Animals were anesthetized in 0.03% tricaine methane sulfonate (MS-222; E1052, Sigma-Aldrich) before undergoing complete transection of the spinal cord at segment 12. The procedure was carried out using a microknife (10318-14, Fine Science Tools) under continuous visual guidance with a stereomicroscope (Leica Microsystem SD9, Leica). After the injury, all animals were transferred to standard freshwater housing conditions supplemented with acetylsalicylic acid (aspirin, N02BA01, Evolan Pharma AB; 2.5 mg/liter) and maintained for 3, 7, 14, or 21 dpi, depending on the experimental time point. The selection of the time points was based on our previous research into neuronal electrophysiological changes after injury in the adult zebrafish spinal cord (*6*). This rationale ensured that early, intermediate, and late postlesion phases were adequately represented, thereby allowing us to capture both the acute responses and the subsequent regenerative processes. In particular, these time points encompass key cellular and molecular events such as the initial injury-induced disruption of neuronal activity, the activation of intrinsic repair mechanisms, and the progressive restoration of functional connectivity. This selection also ensures that critical regenerative phases of SCI are included and facilitates direct comparison with earlier zebrafish studies (*6*, *33*, *38*, *40*, *83*–*85*) while at the same time providing a robust framework for cross-study validation and mechanistic interpretation of neuronal plasticity and recovery.

### Morphological reconstructions

pMNs and dV2a-INs in uninjured animals, along with putative damaged pMNs and dV2a-INs located near the lesion site (0 to 180 μm; injured animals), were passively filled with 0.5 to 1% neurobiotin tracer [NB, N-(2-aminoethyl) biotinamide hydrochloride; SP-1120, Vector Labs] using the same methodology as for electrophysiological recordings (see the “Electrophysiology” section) to post hoc expose and reconstruct their morphologies. Once a whole-cell configuration was achieved, pMNs and dV2a-INs were maintained in the recording chamber for about 30 min and depolarized via current pulses to ensure complete tracer diffusion. The spinal cords were then extracted, thoroughly washed with PBS, and fixed in 4% PFA overnight at 4°C. Afterward, the tissue was incubated with Alexa Fluor–conjugated streptavidin (dilution 1:500; see table S2) overnight at 4°C, protected from light. After thoroughly rinsing with PBS, the tissue was mounted on glass slides using a PBS-based mounting solution containing 80% glycerol (G5516, Sigma-Aldrich).

### PI labeling

To visualize dying cells in the spinal cord after injury, animals were deeply anesthetized with 0.3 to 0.5% tricaine methane sulfonate (MS-222) and dissected in extracellular solution (see the “Electrophysiology” section). The axial musculature and vertebral arches were carefully removed to expose the spinal cord and enhance tissue permeability. Spinal cord preparations were then incubated in an extracellular solution containing 5% PI (P4864, Sigma-Aldrich) for 30 min at room temperature with gentle agitation. Following incubation, tissues were rinsed three times (10 min each) in fresh extracellular solution and subsequently fixed in 4% PFA overnight at 4°C. After fixation, samples were thoroughly washed with PBS and mounted on glass slides using a mounting medium composed of 80% glycerol in PBS.

### Immunohistochemistry

Experiments were conducted in the adult zebrafish spinal cord as previously described (*6*, *36*, *38*, *86*, *87*). Briefly, immunolabeling was performed on spinal whole-mount preparations and cryosections. For cryosectioning, tissue was cryoprotected in 30% sucrose (S7903, Sigma-Aldrich) in PBS at 4°C, embedded in Cryomount (45830, HistoLab), frozen in dry ice–cooled isopentane (277258, Sigma-Aldrich), and stored at −80 C. Transverse sections (20 to 25 μm; CryoStar NX70; 387928, Thermo Fisher Scientific) were used for immunohistochemistry. Tissues were washed in PBS, blocked, and incubated with primary antibodies for 1 to 3 days at 4 C (see table S2). After washing, fluorescent or biotinylated secondary antibodies (see table S2) were applied overnight (4°C). Streptavidin-Alexa was added when biotinylated antibodies were used. Samples were rinsed and mounted on slides. Imaging was performed on a confocal laser scanning microscope, maintaining identical acquisition settings to allow consistent comparisons across conditions.

### WFA histochemistry

To visualize CSPGs in the ECM, we used biotinylated WFA (L1516, Sigma-Aldrich, RRID: AB_2620171), a well-established lectin probe that selectively binds to *N*-acetylgalactosamine residues enriched in perineuronal nets and CSPG-containing structures. Whole-mount spinal cords or cryosections were rinsed three times in PBS to remove residual fixative. The samples were then incubated in blocking, followed by overnight incubation at 4°C with biotinylated WFA (1:500 dilution in blocking solution). After extensive PBS washes to eliminate unbound lectin, fluorescent detection was performed by incubating the samples with Alexa Fluor–conjugated streptavidin (488, 555, or 647 nm), providing a sensitive readout of WFA-bound CSPGs through streptavidin-biotin amplification. Last, tissues were washed in PBS, mounted onto glass slides with mounting medium, and cover-slipped. Imaging was carried out using confocal laser scanning microscopy under identical acquisition settings to ensure reliable comparisons across experimental conditions.

### Electrophysiology

Zebrafish were deeply anesthetized in ice-cold extracellular solution. To expose the spinal cord, the skin, axial muscles, and surrounding bones were carefully removed. The isolated spinal cord was then transferred to a recording chamber continuously perfused with extracellular solution containing 135.2 mM NaCl (S7653, Sigma-Aldrich), 2.9 mM KCl (P9333, Sigma-Aldrich), 2.1 mM CaCl_2_ (C5080, Sigma-Aldrich), 10 mM Hepes (H3375, Sigma-Aldrich), and 10 mM glucose (G7528, Sigma-Aldrich), adjusted to pH 7.4. Whole-cell intracellular recordings from primary MNs and dorsal V2a-INs were performed using current-clamp configurations. Recording electrodes (5 to 7 MΩ) were pulled from borosilicate glass (outer diameter: 1.5 mm; inner diameter: 0.87 mm; 1405063, Hilgenberg) using a micropipette puller (model P-97, Sutter Instruments) and filled with an internal solution composed of 120 mM K-gluconate (P1847, Sigma-Aldrich), 5 mM KCl, 10 mM Hepes, 4 mM adenosine 5′-triphosphate magnesium salt (Mg_2_ATP) (A9187, Sigma-Aldrich), 0.3 mM guanosine 5′-triphosphate sodium salt hydrate (Na_4_GTP) (G8877, Sigma-Aldrich), and 10 mM Na-phosphocreatine (P7936, Sigma-Aldrich), adjusted to pH 7.4. pMNs (retrograde traced) and dV2a-INs (GFP^+^) were visually identified under a microscope (LNscope; Luigs & Neumann) equipped with a charge-coupled device camera (INFINITY2-1RM, Lumenera) at ×60 magnification. Electrode positioning was achieved with a motorized micromanipulator (SM-10, Luigs & Neumann) while maintaining constant positive pressure. Electrical signals were amplified using a MultiClamp 700B amplifier (Molecular Devices). All recordings were conducted at 24° ± 1°C. Electrophysiological properties were measured without injecting any bias current. Only neurons with a stable RMP of −40 mV or lower and less than 5% variation in input resistance were analyzed. EPSPs were detected and analyzed semiautomatically under supervision using AxoGraph (version X 1.5.4; AxoGraph Scientific, Sydney, Australia; RRID: SCR_014284) or Clampfit (version 10.6; Molecular Devices; RRID: SCR_011323). Pharmacological agents were added to the extracellular solution in combination, including NBQX (20 μM; N183, Sigma-Aldrich) and AP5 (50 μM; A5282, Sigma-Aldrich), prepared by diluting stock solutions in distilled water.

### ChABC treatment

To assess the role of the ECM in SCI responses, ECM components were enzymatically degraded using ChABC from *P. vulgaris* (C3667, Sigma-Aldrich). ChABC was prepared in a solution containing 50 mM tris-HCl (T1503, Sigma-Aldrich), 60 mM sodium acetate (S2889, Sigma-Aldrich), and 0.02% bovine serum albumin in PBS, following the manufacturer’s instructions. The same solution without ChABC was used as a vehicle control. Following SCI, animals were maintained in a heated water system at ~30°C for 4 hours to optimize its activity. Fish were then anesthetized, and 1 μl of either vehicle or ChABC (4 or 8 U/ml) was injected directly at the lesion site using a fine glass micropipette under a stereomicroscope. Postinjection, animals were kept in warm water containing acetylsalicylic acid (2.5 mg/liter) for 3 days, after which they were returned to standard freshwater housing until further analysis. Animals were monitored daily for signs of stress or infection.

### Behavioral tests

To assess spontaneous locomotor activity, adult zebrafish were subjected to the open-field test using the ZebraCube system (Viewpoint, Lyon, France). Individual fish were gently transferred into circular petri dishes (14 cm in diameter) filled with system water and placed within the recording arena. Following a habituation period of 12 min to minimize stress-induced artifacts, swim behavior was recorded for 5 min under consistent lighting and environmental conditions. Video recordings were subsequently extracted and analyzed using Noldus EthoVision XT (Noldus, the Netherlands) automated tracking software (see the “Analysis” section). Parameters such as total distance traveled, mean velocity, and mobility state (e.g., active versus inactive periods) were quantified to evaluate locomotor performance. All experiments were performed during the same time window of the day to reduce circadian variability. A separate group of fish was subjected to a modified forced swim test using a 5-liter swim tunnel respirometer (SW10050, Loligo Systems) to evaluate swim performance under increasing water flow conditions. Fish were individually introduced into the swim tunnel and allowed to acclimate for 2 min at a baseline flow velocity of 141 rpm (rotations per minute), corresponding to a mild current that encouraged steady swimming without inducing fatigue. Subsequently, the water flow velocity was increased by 23.5 rpm every 30 s until the fish could no longer maintain their position against the current and were pushed to the rear of the chamber. The latency to exhaustion, defined as the time at which the fish ceased forward swimming and was unable to resist the flow, was recorded and used as an index of swim performance.

### Analysis

Fluorescent imaging of whole-mount adult zebrafish spinal cord preparations and cryosections was performed using a Zeiss LSM800 laser scanning confocal microscope equipped with a 20× dry or 40× oil-immersion objective. For each whole-mount spinal cord hemisegment analyzed, z-stacks were acquired with a step size of 1 to 4 μm, to ensure comprehensive coverage of the hemisegment. Most images presented were generated from selected subsets of the original z-stacks, focusing on regions near the pMN and dV2a-IN populations (medial motor column), to enhance the visualization and presentation of the data. Colocalization was assessed by visually identifying structures in merged images that displayed a color indicative of the overlapping signal from different fluorescent antibodies or markers. Neuronal morphological features assessed included soma area (soma size, in square micrometers) and the rostrocaudal extension of neurites, encompassing both dendrites and axons. All morphological measurements, as well as cell quantification, were performed using ImageJ software (National Institutes of Health, https://imagej.net/ij/; RRID: SCR_003070). Sholl analysis was conducted with the Sholl Analysis plugin for ImageJ to evaluate the complexity of neurite arborization. In whole-mount spinal cord preparations, the relative position of spinal neurons was determined using anatomical landmarks, including the lateral, dorsal, and ventral edges of the spinal cord, as well as the central canal, using ImageJ. The average fluorescence intensity (mean gray value) of WFA labeling was measured using ImageJ within manually drawn regions of interest (ROIs). ROIs were placed either in the medial motor column, where pMNs and dV2a-INs are located, or around individual neuronal somata (within a 2- to 3-μm perimeter) using a z-stack frame representing the neuronal epicenter, typically corresponding to the largest cross-sectional area of the cell body. For analysis of fluorescence intensity of α-tubulin and V2a-INs (*chx10:GFP*) during regeneration, measurements were performed on images generated by merging the entire z-stack. ROIs in these cases encompassed the injury site to ensure representative sampling of the regenerating area. The average intensity from an unstained image region was subtracted to correct for differences in background fluorescence levels. For comparative analysis of WFA fluorescence intensity across experimental conditions, background-corrected images were pseudo-colored using the “Fire” lookup table in ImageJ to enhance visual interpretation. For the analysis of SV2^+^ presynaptic sites, high-resolution images were generated by merging three consecutive optical slices from the z-stack at the level of the neuronal soma epicenter, typically corresponding to the largest cross-sectional area of the cell body. The entire merged image was subsequently analyzed without the use of predefined ROIs, thereby ensuring unbiased quantification. Quantification of SV2^+^ puncta was performed using the Particle Analysis tool in ImageJ. Intensity thresholds were carefully and consistently defined manually, after which only the particles located in close proximity or in direct contact with the pMN surface were considered and counted (manually). This combined semiautomated and manual approach enabled a reliable and reproducible measurement of both the number and spatial distribution of presynaptic puncta at the pMN somata. All presented images were prepared using Adobe Photoshop (Adobe Systems Inc., San Jose, CA, USA; RRID: SCR_014199). To enhance data visualization, some fluorescent images were inverted, displayed as single channels, or converted to magenta-green color schemes to improve accessibility for color-blind readers. Digital adjustments to brightness and contrast were applied uniformly to avoid distortion of biological information. Because of the three-dimensional structure of whole-mount spinal cord preparations, pMNs and dV2a-INs are often located in different focal planes, which can result in misleading visual representations when entire hemisegments are shown. To ensure accurate visualization and quantification, images used for data extraction were selected from distinct focal planes, typically at the midline, where the neuronal soma displays its largest diameter, or compiled from a subset of optical slices spanning from the most lateral edge of the soma to the midline. All electrophysiological parameters were measured and analyzed using Clampfit software (version 11.0; Molecular Devices). EPSP amplitude was calculated as the difference between the baseline membrane potential and the peak of the event. EPSP frequency was assessed from 2-s-long recordings. The adaptation index was calculated on the basis of the firing duration during a 500-ms depolarizing current injection at 120% of the rheobase. PCA was performed using nine electrophysiological parameters to reduce the dataset’s dimensionality while preserving the most important variance. The first two principal components were plotted to visualize the variability within the data, facilitating the identification of underlying patterns and potential groupings among samples. The regeneration index (RI) was calculated as the proportion of bridge formation spanning the injury site relative to the dorsoventral axis of adjacent uninjured spinal cord segments. Bridging coverage of 5% or less was considered negligible and assigned an RI value of 0. Data normalization was performed individually for each parameter, either relative to the highest observed value for that specific feature or scaled to its minimum and maximum values, as indicated in heatmaps. For behavioral analysis, parameters including average velocity, total distance traveled, immobility ratio, and additional metrics were extracted using EthoVision XT automated tracking software (Version 17, Noldus, the Netherlands; RRID: SCR_000441). All figures and graphs were prepared using Adobe Illustrator (Adobe Systems Inc., San Jose, CA, USA; RRID: SCR_010279), GraphPad Prism (GraphPad Software Inc.; RRID: SCR_002798), and Microsoft Excel (used for spider plots; RRID: SCR_016137).

### Reproducibility

To minimize observer, selection, and confirmation biases, multiple strategies were implemented throughout the experimental and analytical processes. First, data analysis was performed by an individual (analyst) who was independent from the person who collected the data (experimenter), using a predefined, unbiased, computer-assisted image analysis workflow as described above. Second, key experiments were independently repeated by two or more individuals to ensure reproducibility. Third, all experimental conditions included large cohorts of animals across multiple replicated experiments to enhance statistical robustness and generalizability. Despite these independent approaches, all individuals involved reached the same conclusions, underscoring the reliability and reproducibility of the findings. In addition, blinding procedures were not implemented in the present study due to the nature of the experimental design. Electrophysiological recordings, anatomical analyses, and cellular assessments were performed near the lesion site, requiring the experimenter to identify the injured region in each subject. Moreover, injured animals displayed significantly altered motor behavior compared to uninjured controls, and during regeneration analyses, the extent of spinal cord bridging reveals the approximate timing postinjury. These considerations rendered complete blinding infeasible; however, all analyses were conducted using standardized and consistent protocols to minimize potential bias. Last, no exclusion criteria were preestablished, and all data obtained are presented in the analyses.

### Statistics

Parametric tests, including the two-tailed (two-sided) unpaired or paired Student’s *t* test and one-way analysis of variance (ANOVA; ordinary), were used to analyze the statistical significance of mean differences among the experimental groups. Post hoc Tukey’s test complemented this analysis for multiple comparisons between all groups, as well as Dunnett’s test for comparisons against a control group. All statistical analyses, including the PCA, were performed using Prism (GraphPad Software Inc.). In the graphs presented, the significance levels are marked as *P < 0.05, **P < 0.01, ***P < 0.001, and ****P < 0.0001, while n.s. indicates no significance. The data are displayed as mean ± SEM, mean ± SD, normalized heatmaps, floating bars representing mean and minimum-maximum values, bar graphs illustrating mean ± SEM, and violin plots. In addition, box plots are presented, showing median, 25th, and 75th percentiles (depicted as boxes and lines), along with minimum and maximum values indicated by whiskers. The *n* values indicate the total count of validated animals per group, cells, or events, as outlined in table S1. Most experiments were conducted independently by different investigators.
