# Supplementary material for: Time-dependent adaptations of damaged neurons and their microenvironment in the regenerating adult zebrafish spinal cord
Source: Sci Adv. 2026 Mar 6;12(10):eaea2882. doi: 10.1126/sciadv.aea2882 (PMC12965314; doi:10.1126/sciadv.aea2882)
Supplement: Supplementary file 1 — Figs. S1 to S6 Tables S1 and S2 [file sciadv.aea2882_sm.pdf]

Science Advances
AAAS

Supplementary Materials for

Time-dependent adaptations of damaged neurons and their microenvironment in the regenerating adult zebrafish spinal cord

Leslie Lafouasse *et al.*

Corresponding author: Konstantinos Ampatzis, konstantinos.ampatzis@ki.se

Sci. Adv. **12**, eaea2882 (2026)
DOI: 10.1126/sciadv.aea2882

This PDF file includes:

Figs. S1 to S6
Tables S1 and S2

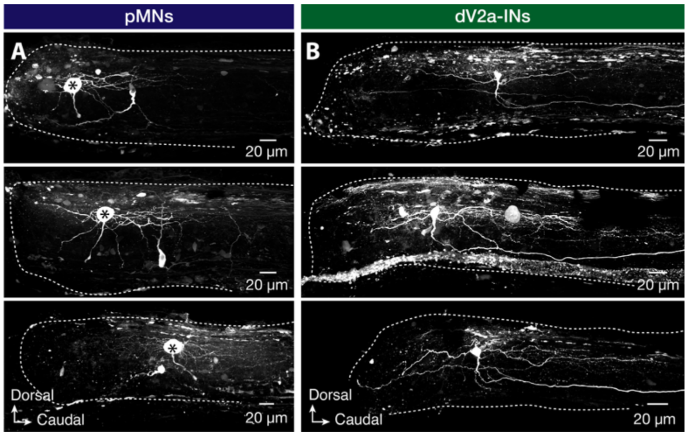

Fig. S1. Morphological features of damaged pMNs and dV2a-INs in zebrafish spinal cord. (**A-B**) Microphotographs showing representative examples of neurobiotin-filled pMNs and dV2a-INs close to the injury site. dV2a-IN, dorsal V2a interneuron; pMN, primary motoneuron; Asterisks indicate the pMN cell bodies.

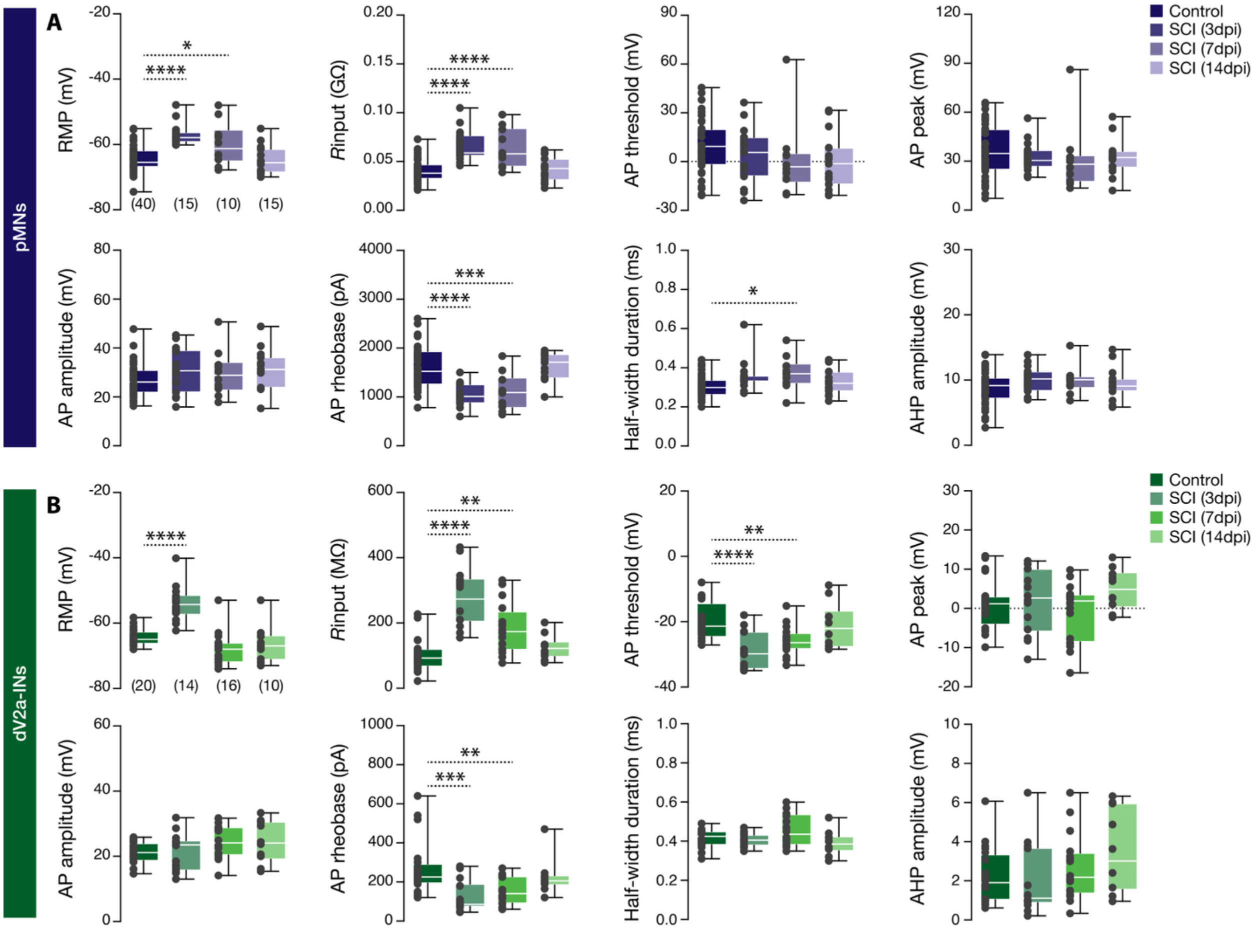

Fig. S2. Physiological properties of the adult zebrafish putatively damaged pMNs and dV2a-INs. (**A-B**) Detailed analysis of the electrical properties of the pMNs and dV2a-INs in uninjured animals and those post-injury (3, 7, and 14 dpi). AHP, after hyperpolarization potential; AP, action potential; dpi, days post-injury; dV2a-IN, dorsal V2a interneuron; pMN, primary motoneuron; Rheo, rheobase; *R*input, input resistance; RMP, resting membrane potential; SCI, spinal cord injury. Data are presented as box plots showing the median with 25/75 percentile (box and line) and minimum–maximum (whiskers). **P* < 0.05; ***P* < 0.01; ****P* < 0.001; *****P* < 0.0001. For detailed statistics, see table S1.

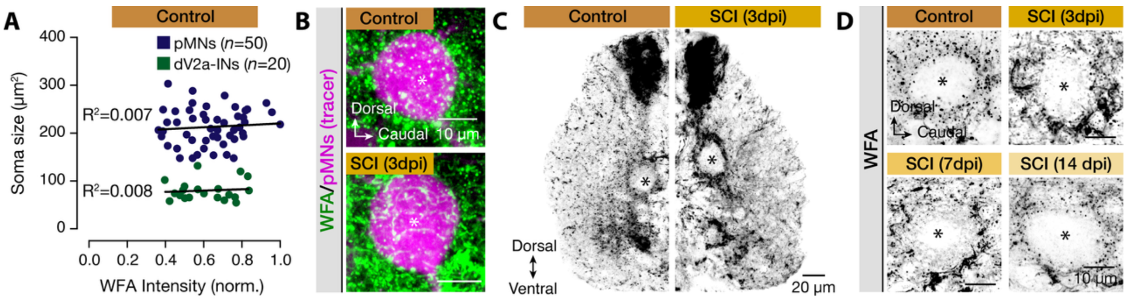

Fig. S3. ECM structural remodeling in the adult zebrafish spinal cord. (**A**) The normalized WFA intensity in relation to pMN and dV2a-IN soma size in uninjured animals. (**B**) Confocal images from a stack of optical sections display the WFA staining (green) surrounding the pMN (magenta) soma in control and at 3dpi. (**C**) Inverted fluorescent images of WFA staining obtained from coronal spinal cord sections. (**D**) Inverted fluorescent images demonstrate the WFA structural changes near the pMN soma following SCI. Asterisks indicate the pMN cell bodies. dpi, days post-injury; dV2a-IN, dorsal V2a interneuron; pMN, primary motoneuron; SCI, spinal cord injury; WFA, Wisteria floribunda agglutinin. For detailed statistics, see table S1.

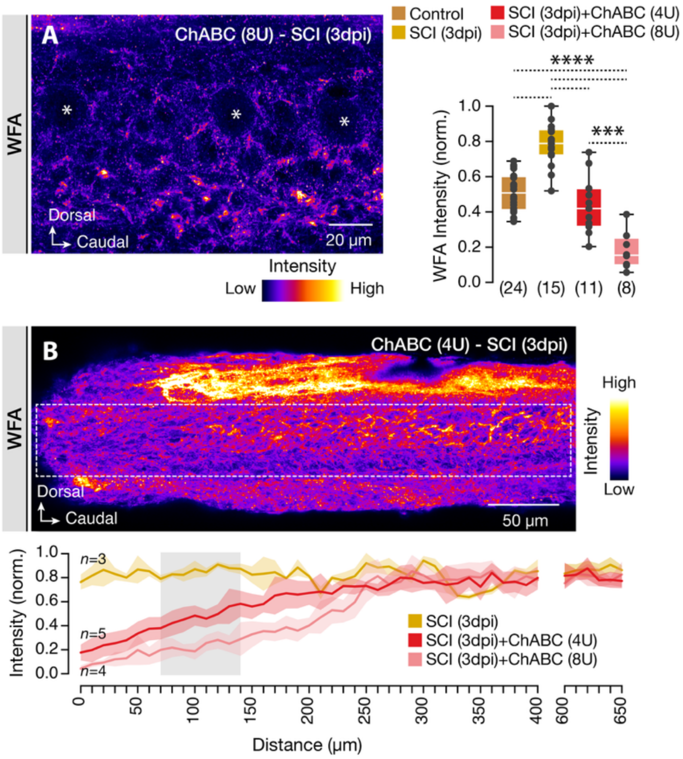

Fig. S4. Dose and spatial controlled digestion of ECM by chondroitinase ABC (ChABC). (**A**) Pseudo-colored image of WFA intensity from the whole mount spinal cord after the administration of 8 units of ChABC. Analysis of WFA intensity at 3 dpi following enzymatic digestion of ECM structures using 4 and 8 units of chondroitinase ABC. (**B**) Pseudo-colored image of the spatial WFA intensity in the adult zebrafish motor column (dashed box) following the administration of 4 units of chondroitinase ABC at the lesion site. Plot depicting the spatial WFA intensity in relation to the distance from the lesion/injection site after ChABC at 3 dpi. Asterisks indicate the pMN cell bodies. ChABC, Chondroitinase ABC; dpi, days post-injury; SCI, spinal cord injury; WFA, Wisteria floribunda agglutinin. Data are presented as mean ± S.D., and as box plots showing the median with 25/75 percentiles (box and line) and minimum–maximum (whiskers). ***$P < 0.001$; ****$P < 0.0001$. For detailed statistics, see table S1.

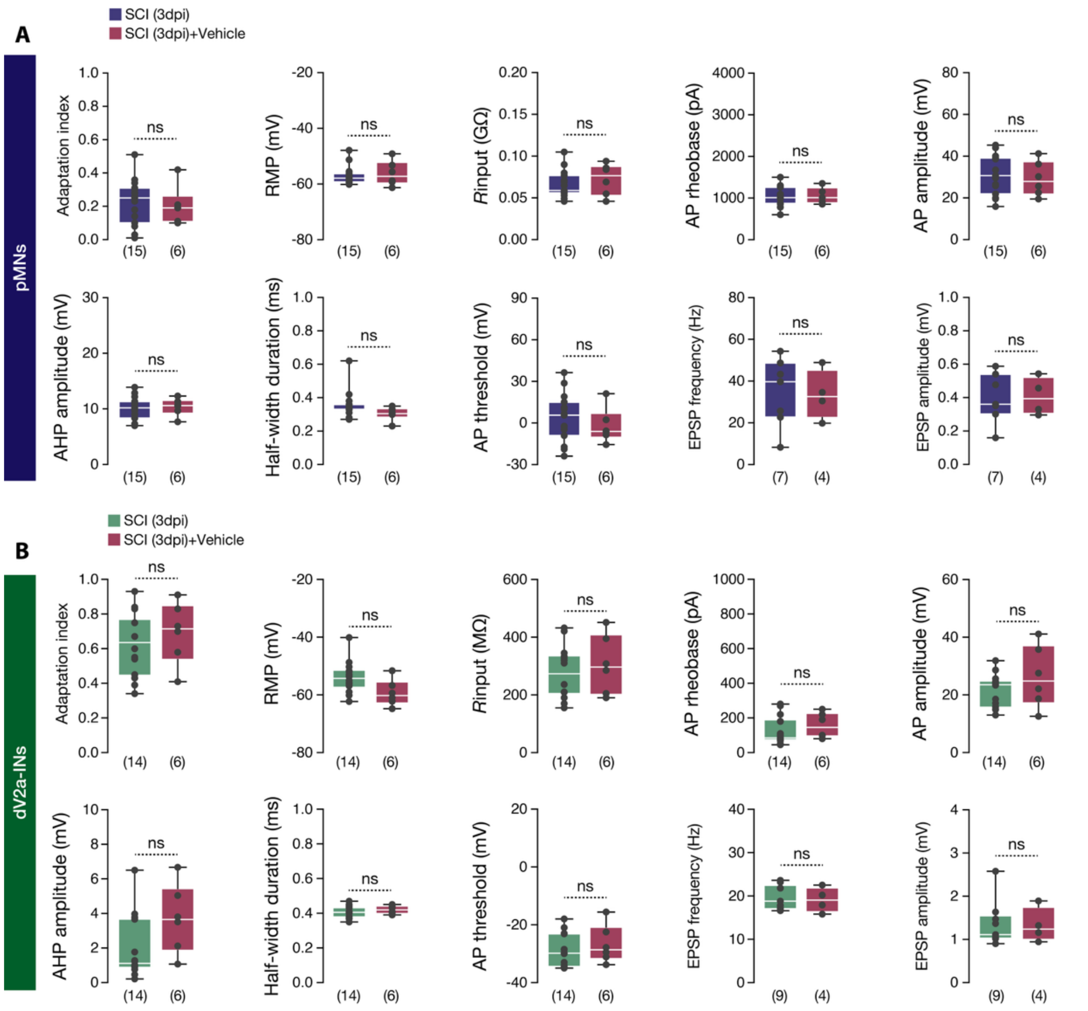

Fig. S5. Electrical and synaptic properties of pMNs and dV2a-INs following injection of the vehicle control at 3 dpi. (**A-B**) Detailed analysis of the electrical properties of the pMNs and dV2a-INs in 3 dpi animals and those at 3 dpi that receive the vehicle control for the ChABC-related experiments. AHP, after hyperpolarization potential; AP, action potential; dpi, days post-injury; EPSP, excitatory postsynaptic potential; *R*input, input resistance; RMP, resting membrane potential. Data are presented as box plots showing the median with 25/75 percentile (box and line) and minimum–maximum (whiskers). ns, not significant. For detailed statistics, see table S1.

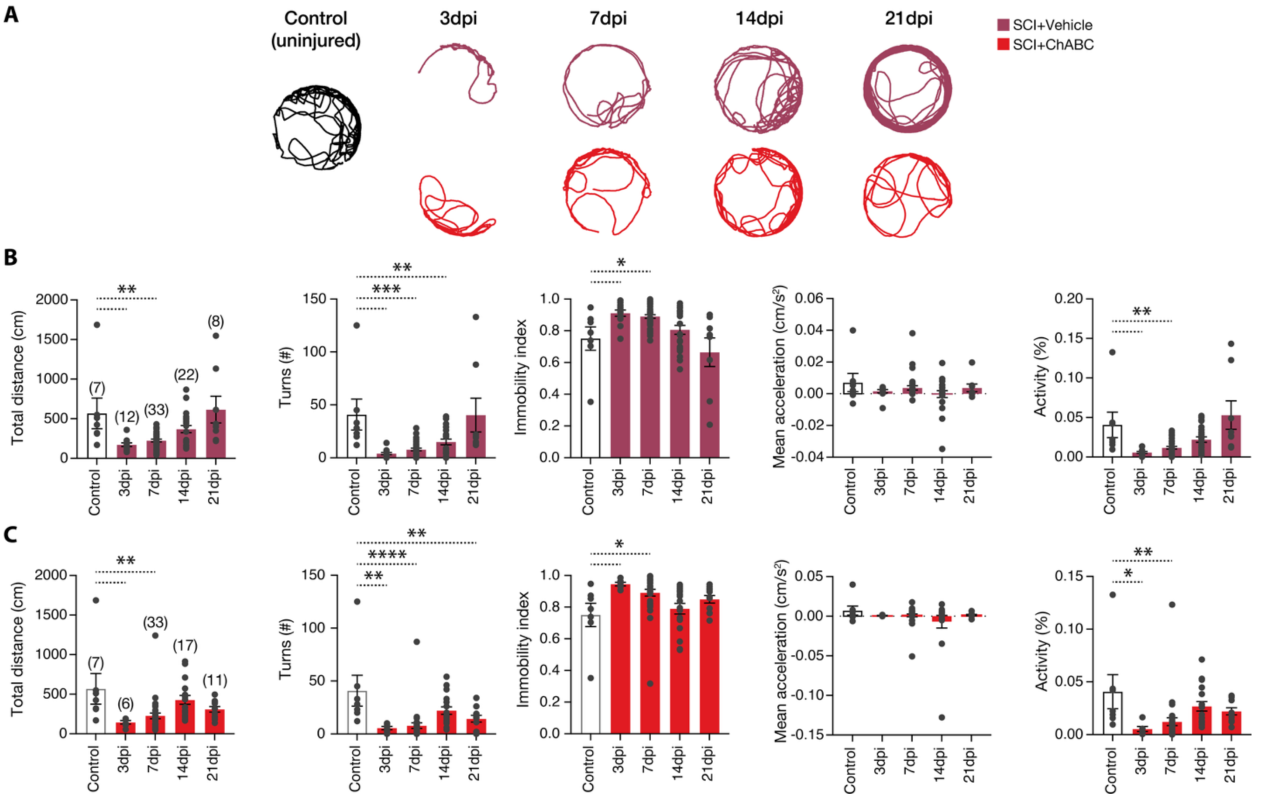

Fig. S6. Behavioral parameters of the adult zebrafish during regeneration. (**A**) Representative traces of uninjured and ChABC-untreated (SCI) and ChABC-treated (SCI+ChABC) injured zebrafish swimming in the open field test during regeneration. (**B-C**) Analysis of behavioral parameters obtained at different time points during spinal cord regeneration and comparison with the control (uninjured) animals. ChABC, Chondroitinase ABC; dpi, days post-injury; SCI, spinal cord injury. Data are presented as bar graphs (mean ± s.e.m.), and as box plots showing the median with 25/75 percentiles (box and line) and minimum–maximum (whiskers). *$P < 0.05$; **$P < 0.01$; ***$P < 0.001$; ****$P < 0.0001$. For detailed statistics, see table S1.

Table S1. Detailed statistics

Figure	Statistics	Result	Post-hoc Test	comparison	Significance	*P-value*
**MAIN FIGURES**						
**1B**	Unpaired *t*-test	t = 15.34, df = 378 (Two-tailed)		MNs<br>pMNs	****	$P < 0.0001$
	Unpaired *t*-test	t = 10.53, df = 485 (Two-tailed)		V2a-INs<br>dV2a-INs	****	$P < 0.0001$
	Descriptive	MNs (*n* = 343 neurons): 74.87 ± 2.9<br>pMNs (*n* = 37 neurons): 218.3 ± 6.7<br>V2a-INs (*n* = 465 neurons): 40.43 ± 0.9<br>dV2a-INs (*n* = 22 neurons): 89.47 ± 6.4				
**1G (pMNs)**	Unpaired *t*-test	t = 5.186, df = 13 (Two-tailed)		Uninjured<br>Injured	***	$P = 0.0002$
	Descriptive	Uninjured (*n* = 9 zebrafish): 209.8 ± 7.0<br>Injured (*n* = 6 zebrafish): 111.9 ± 20.8				
**1H (dV2a-INs)**	Unpaired *t*-test	t = 6.926, df = 14 (Two-tailed)		Uninjured<br>Injured	****	$P < 0.0001$
	Descriptive	Uninjured (*n* = 11 zebrafish): 238.4 ± 9.7<br>Injured (*n* = 5 zebrafish): 116.1 ± 15.3				
**1I (pMNs)**	Unpaired *t*-test	t = 0.85, df = 58 (Two-tailed)		Control<br>SCI (7dpi)	ns	$P = 0.3966$
	Descriptive	Control (*n* = 34 neurons): 211 ± 5.7<br>SCI (7dpi) (*n* = 26 neurons): 219 ± 7.7				
**1I (dV2a-INs)**	Unpaired *t*-test	t = 0.19, df = 45 (Two-tailed)		Control<br>SCI (7dpi)	ns	$P = 0.8458$
	Descriptive	Control (*n* = 22 neurons): 89.47 ± 6.4<br>SCI (7dpi) (*n* = 25 neurons): 91.1 ± 5.4				
**2B**	Unpaired *t*-test	t = 3.86, df = 13 (Two-tailed)		Control<br>SCI (3dpi)	**	$P = 0.0019$
	Descriptive	Control (*n* = 8 zebrafish): 322.3 ± 15.4<br>SCI (3dpi) (*n* = 7 zebrafish): 390.7 ± 6.7				
**2D (pMNs)**	Unpaired *t*-test	t = 9.07, df = 19 (Two-tailed)		Control<br>SCI (3dpi)	****	$P < 0.0001$
	Descriptive	Control (*n* = 8 zebrafish): 10.88 ± 3.4<br>SCI (3dpi) (*n* = 13 zebrafish): 71.12 ± 4.7				
**2D (dV2a-INs)**	Unpaired *t*-test	t = 2.802, df = 18 (Two-tailed)		Control<br>SCI (3dpi)	*	$P = 0.0118$
	Descriptive	Control (*n* = 8 zebrafish): 57.92 ± 6.1<br>SCI (3dpi) (*n* = 12 zebrafish): 78.89 ± 4.5				
**2G (pMNs)**	One-way ANOVA	$F_{(3, 76)} = 161$<br>$P < 0.0001$	Dunnett's test	Control<br>SCI (3dpi)	****	$P_{adj} < 0.0001$
				Control<br>SCI (7dpi)	****	$P_{adj} < 0.0001$
				Control<br>SCI (14dpi)	ns	$P_{adj} = 0.8652$
	Descriptive	Control (*n* = 40 neurons): 0.87 ± 0.01<br>SCI (3dpi) (*n* = 15 neurons): 0.22 ± 0.03<br>SCI (7dpi) (*n* = 10 neurons): 0.68 ± 0.03<br>SCI (14dpi) (*n* = 15 neurons): 0.85 ± 0.01				
**2G (dV2a-INs)**	One-way ANOVA	$F_{(3, 56)} = 7.89$<br>$P = 0.0002$	Dunnett's test	Control<br>SCI (3dpi)	***	$P_{adj} = 0.0007$
				Control<br>SCI (7dpi)	**	$P_{adj} = 0.0012$
				Control<br>SCI (14dpi)	ns	$P_{adj} = 0.9344$
	Descriptive	Control (*n* = 20 neurons): 0.82 ± 0.02<br>SCI (3dpi) (*n* = 14 neurons): 0.62 ± 0.04<br>SCI (7dpi) (*n* = 16 neurons): 0.63 ± 0.04<br>SCI (14dpi) (*n* = 10 neurons): 0.79 ± 0.04				
**3A (frequency)**	One-way ANOVA	$F_{(3, 42)} = 9.989$<br>$P < 0.0001$	Dunnett's test	Control<br>SCI (3dpi)	***	$P_{adj} = 0.0002$
				Control<br>SCI (7dpi)	***	$P_{adj} = 0.0003$
				Control<br>SCI (14dpi)	ns	$P_{adj} = 0.0605$
	Descriptive	Control (*n* = 22 neurons): 17.14 ± 1.18<br>SCI (3dpi) (*n* = 7 neurons): 34.67 ± 6.17<br>SCI (7dpi) (*n* = 9 neurons): 32.67 ± 2.4<br>SCI (14dpi) (*n* = 8 neurons): 26.22 ± 3.98				

**3A (amplitude)**	One-way ANOVA	$F_{(3, 42)}$ = 5.776 P = 0.0021	Dunnett's test	Control SCI (3dpi)	**	P_{adj} = 0.0072
				Control SCI (7dpi)	**	P_{adj} = 0.0045
				Control SCI (14dpi)	ns	P_{adj} = 0.4112
	Descriptive	Control (*n* = 22 neurons): 0.25 ± 0.01<br>SCI (3dpi) (*n* = 7 neurons): 0.39 ± 0.05<br>SCI (7dpi) (*n* = 9 neurons): 0.39 ± 0.04<br>SCI (14dpi) (*n* = 8 neurons): 0.31 ± 0.02				
**3B (frequency)**	One-way ANOVA	$F_{(3, 33)}$ = 13.95 P < 0.0001	Dunnett's test	Control SCI (3dpi)	****	P_{adj} <0.0001
				Control SCI (7dpi)	*	P_{adj} = 0.0469
				Control SCI (14dpi)	ns	P_{adj} = 0.4901
	Descriptive	Control (*n* = 10 neurons): 12.5 ± 1.01<br>SCI (3dpi) (*n* = 9 neurons): 19.73 ± 0.94<br>SCI (7dpi) (*n* = 11 neurons): 15.9 ± 1.1<br>SCI (14dpi) (*n* = 7 neurons): 10.63 ± 0.83				
**3B (amplitude)**	One-way ANOVA	$F_{(3, 33)}$ = 8.494 P = 0.0003	Dunnett's test	Control SCI (3dpi)	**	P_{adj} = 0.001
				Control SCI (7dpi)	*	P_{adj} = 0.0108
				Control SCI (14dpi)	ns	P_{adj} = 0.9862
	Descriptive	Control (*n* = 10 neurons): 0.67 ± 0.05<br>SCI (3dpi) (*n* = 9 neurons): 1.35 ± 0.17<br>SCI (7dpi) (*n* = 11 neurons): 1.17 ± 0.12<br>SCI (14dpi) (*n* = 7 neurons): 0.62 ± 0.09				
**3C (frequency)**	Paired *t*-test	t = 10.05, df = 5 (Two-tailed)		Saline NBQX+AP5	***	P = 0.0002
	Descriptive	Saline (*n* = 6 neurons): 34.66 ± 3.4<br>NBQX+AP5 (*n* = 6 neurons): 8.11 ± 0.8				
**3C (amplitude)**	Paired *t*-test	t = 4.259, df = 5 (Two-tailed)		Saline NBQX+AP5	**	P = 0.008
	Descriptive	Saline (*n* = 6 neurons): 0.42 ± 0.03<br>NBQX+AP5 (*n* = 6 neurons): 0.21 ± 0.05				
**3C (RMP)**	Paired *t*-test	t = 4.44, df = 5 (Two-tailed)		Saline NBQX+AP5	**	P = 0.0068
	Descriptive	Saline (*n* = 6 neurons): -55.38 ± 1.76<br>NBQX+AP5 (*n* = 6 neurons): -62.38 ± 2.42				
**3D (frequency)**	Paired *t*-test	t = 44.62, df = 3 (Two-tailed)		Saline NBQX+AP5	**	P = 0.0014
	Descriptive	Saline (*n* = 4 neurons): 19.7 ± 1.86<br>NBQX+AP5 (*n* = 4 neurons): 4.59 ± 0.58				
**3D (amplitude)**	Paired *t*-test	t = 7.653, df = 3 (Two-tailed)		Saline NBQX+AP5	**	P = 0.0046
	Descriptive	Saline (*n* = 4 neurons): 1.27 ± 0.17<br>NBQX+AP5 (*n* = 4 neurons): 0.37 ± 0.15				
**3D (RMP)**	Paired *t*-test	t = 3.372, df = 3 (Two-tailed)		Saline NBQX+AP5	*	P = 0.0433
	Descriptive	Saline (*n* = 4 neurons): -53.1 ± 1.78<br>NBQX+AP5 (*n* = 4 neurons): -61.3 ± 1.1				
**3F**	Descriptive	$SV2^+$/V2a-INs^+ (*n* = 4 zebrafish): 27.5 ± 1.69				
**3G**	One-way ANOVA	$F_{(3, 52)}$ = 4.668 P = 0.0058	Dunnett's test	Control SCI (3dpi)	ns	P_{adj} = 0.3492
				Control SCI (7dpi)	**	P_{adj} = 0.0025
				Control SCI (14dpi)	ns	P_{adj} = 0.897
	Descriptive	Control (*n* = 17 zebrafish): 21.8 ± 1.17<br>SCI (3dpi) (*n* = 9 zebrafish): 19.01 ± 1.42<br>SCI (7dpi) (*n* = 18 zebrafish): 16.25 ± 1.0<br>SCI (14dpi) (*n* = 12 zebrafish): 20.79 ± 1.46				
**4D**	One-way ANOVA	$F_{(3, 69)}$ = 19.56 P < 0.0001	Dunnett's test	Control SCI (3dpi)	****	P_{adj} <0.0001
				Control SCI (7dpi)	**	P_{adj} = 0.0068
				Control SCI (14dpi)	ns	P_{adj} = 0.9798

	Descriptive	Control (*n* = 24 zebrafish): 0.51 ± 0.02<br>SCI (3dpi) (*n* = 15 zebrafish): 0.78 ± 0.03<br>SCI (7dpi) (*n* = 21 zebrafish): 0.61 ± 0.02<br>SCI (14dpi) (*n* = 13 zebrafish): 0.52 ± 0.03				
**4E**	One-way ANOVA	$F_{(3, 49)}$ = 15.15<br>*P* < 0.0001	Dunnett's test	Control<br>SCI (3dpi)	****	P_{adj} <0.0001
				Control<br>SCI (7dpi)	*	P_{adj} = 0.047
				Control<br>SCI (14dpi)	ns	P_{adj} = 0.7327
	Descriptive	Control (*n* = 18 zebrafish): 0.53 ± 0.02<br>SCI (3dpi) (*n* = 11 zebrafish): 0.76 ± 0.03<br>SCI (7dpi) (*n* = 13 zebrafish): 0.63 ± 0.03<br>SCI (14dpi) (*n* = 11 zebrafish): 0.49 ± 0.02				
**4F**	One-way ANOVA	$F_{(3, 41)}$ = 5.032<br>*P* = 0.0046	Dunnett's test	Control<br>SCI (3dpi)	**	P_{adj} = 0.0057
				Control<br>SCI (7dpi)	ns	P_{adj} = 0.0703
				Control<br>SCI (14dpi)	ns	P_{adj} = 0.9998
	Descriptive	Control (*n* = 14 zebrafish): 0.54 ± 0.02<br>SCI (3dpi) (*n* = 10 zebrafish): 0.69 ± 0.04<br>SCI (7dpi) (*n* = 13 zebrafish): 0.64 ± 0.03<br>SCI (14dpi) (*n* = 8 zebrafish): 0.54 ± 0.02				
**5B**	One-way ANOVA	$F_{(3, 58)}$ = 26.76<br>*P* < 0.0001	Tukey's test	Control<br>SCI (3dpi)	****	P_{adj} <0.0001
				Control<br>SCI (3dpi)+Vehicle	****	P_{adj} <0.0001
				Control<br>SCI (3dpi)+ChABC	ns	P_{adj} = 0.3948
				SCI (3dpi)<br>SCI (3dpi)+Vehicle	ns	P_{adj} = 0.9962
				SCI (3dpi)<br>SCI (3dpi)+ChABC	****	P_{adj} <0.0001
				SCI (3dpi)+Vehicle<br>SCI (3dpi)+ChABC	****	P_{adj} <0.0001
	Descriptive	Control (*n* = 24 zebrafish): 0.51 ± 0.02<br>SCI (3dpi) (*n* = 15 zebrafish): 0.78 ± 0.03<br>SCI (3dpi)+Vehicle (*n* = 12 zebrafish): 0.76 ± 0.03<br>SCI (3dpi)+ChABC (*n* = 11 zebrafish): 0.43 ± 0.04				
**5C (pMN - adaptation index)**	Unpaired *t*-test	t = 0.5, df = 21 (Two-tailed)		SCI (3dpi)<br>SCI (3dpi)+ChABC	ns	*P* = 0.618
	Descriptive	SCI (3dpi) (*n* = 15 neurons): 0.22 ± 0.03<br>SCI (3dpi)+ChABC (*n* = 8 neurons): 0.26 ± 0.06				
**5C (pMN - RMP)**	Unpaired *t*-test	t = 2.824, df = 22 (Two-tailed)		SCI (3dpi)<br>SCI (3dpi)+ChABC	**	*P* = 0.0099
	Descriptive	SCI (3dpi) (*n* = 15 neurons): -56.98 ± 0.86<br>SCI (3dpi)+ChABC (*n* = 9 neurons): -52.5 ± 1.46				
**5C (pMN - EPSP frequency)**	Unpaired *t*-test	t = 2.17, df = 13 (Two-tailed)		SCI (3dpi)<br>SCI (3dpi)+ChABC	*	*P* = 0.0491
	Descriptive	SCI (3dpi) (*n* = 7 neurons): 34.67 ± 6.17<br>SCI (3dpi)+ChABC (*n* = 8 neurons): 50.49 ± 4.18				
**5C (pMN - EPSP amplitude)**	Unpaired *t*-test	t = 0.144, df = 13 (Two-tailed)		SCI (3dpi)<br>SCI (3dpi)+ChABC	ns	*P* = 0.8873
	Descriptive	SCI (3dpi) (*n* = 7 neurons): 0.39 ± 0.05<br>SCI (3dpi)+ChABC (*n* = 8 neurons): 0.4 ± 0.04				
**5C (dV2a-IN - adaptation index)**	Unpaired *t*-test	t = 1.052, df = 15 (Two-tailed)		SCI (3dpi)<br>SCI (3dpi)+ChABC	ns	*P* = 0.3094
	Descriptive	SCI (3dpi) (*n* = 12 neurons): 0.66 ± 0.04<br>SCI (3dpi)+ChABC (*n* = 5 neurons): 0.57 ± 0.04				
**5C (dV2a-IN - RMP)**	Unpaired *t*-test	t = 0.145, df = 15 (Two-tailed)		SCI (3dpi)<br>SCI (3dpi)+ChABC	ns	*P* = 0.886
	Descriptive	SCI (3dpi) (*n* = 12 neurons): -53.19 ± 1.6<br>SCI (3dpi)+ChABC (*n* = 5 neurons): -52.74 ± 2.8				
**5C (dV2a-IN - EPSP frequency)**	Unpaired *t*-test	t = 1.055, df = 12 (Two-tailed)		SCI (3dpi)<br>SCI (3dpi)+ChABC	ns	*P* = 0.312
	Descriptive	SCI (3dpi) (*n* = 9 neurons): 19.73 ± 0.9<br>SCI (3dpi)+ChABC (*n* = 5 neurons): 22.12 ± 2.5				
**5C (dV2a-IN - EPSP amplitude)**	Unpaired *t*-test	t = 0.435, df = 12 (Two-tailed)		SCI (3dpi)<br>SCI (3dpi)+ChABC	ns	*P* = 0.6711
	Descriptive	SCI (3dpi) (*n* = 9 neurons): 1.35 ± 0.1<br>SCI (3dpi)+ChABC (*n* = 5 neurons): 1.47 ± 0.1				

5D	Unpaired *t*-test	t = 4.766, df = 13 (Two-tailed)	SCI (3dpi)<br>SCI (3dpi)+ChABC	***	*P* = 0.0004
	Descriptive	SCI (3dpi) (*n* = 9 zebrafish): 19.01 ± 1.4<br>SCI (3dpi)+ChABC (*n* = 6 zebrafish): 28.98 ± 1.3			
	Unpaired *t*-test	t = 5.601, df = 25 (Two-tailed)	SCI (7dpi)<br>SCI (7dpi)+ChABC	****	*P* < 0.0001
	Descriptive	SCI (7dpi) (*n* = 18 zebrafish): 16.25 ± 1.0<br>SCI (7dpi)+ChABC (*n* = 9 zebrafish): 25.54 ± 1.2			
	Unpaired *t*-test	t = 0.54, df = 16 (Two-tailed)	SCI (14dpi)<br>SCI (14dpi)+ChABC	ns	*P* = 0.5962
	Descriptive	SCI (14dpi) (*n* = 12 zebrafish): 20.87 ± 1.4<br>SCI (14dpi)+ChABC (*n* = 6 zebrafish): 19.51 ± 2.0			
**5E (regeneration)**	Unpaired *t*-test	t = 3.295, df = 44 (Two-tailed)	SCI (7dpi)+Vehicle<br>SCI (7dpi)+ChABC	**	*P* = 0.0019
	Descriptive	SCI (7dpi)+Vehicle (*n* = 22 zebrafish): 0.1 ± 0.03<br>SCI (7dpi)+ChABC (*n* = 24 zebrafish): 0.29 ± 0.04			
	Unpaired *t*-test	t = 2.008, df = 30 (Two-tailed)	SCI (14dpi)+Vehicle<br>SCI (14dpi)+ChABC	ns	*P* = 0.0537
	Descriptive	SCI (14dpi)+Vehicle (*n* = 14 zebrafish): 0.66 ± 0.05<br>SCI (14dpi)+ChABC (*n* = 18 zebrafish): 0.53 ± 0.03			
	Unpaired *t*-test	t = 2.963, df = 20 (Two-tailed)	SCI (21dpi)+Vehicle<br>SCI (21dpi)+ChABC	**	*P* = 0.0077
	Descriptive	SCI (21dpi)+Vehicle (*n* = 10 zebrafish): 0.74 ± 0.04<br>SCI (21dpi)+ChABC (*n* = 12 zebrafish): 0.52 ± 0.05			
**5E (bridging initiation)**	Unpaired *t*-test	t = 2.811, df = 44 (Two-tailed)	SCI (7dpi)+Vehicle<br>SCI (7dpi)+ChABC	**	*P* = 0.0074
	Descriptive	SCI (7dpi)+Vehicle (*n* = 22 zebrafish): 0.31 ± 1.0<br>SCI (7dpi)+ChABC (*n* = 24 zebrafish): 0.7 ± 0.09			
**5F**	Unpaired *t*-test	t = 1.722, df = 15 (Two-tailed)	SCI (7dpi)+Vehicle<br>SCI (7dpi)+ChABC	ns	*P* = 0.1057
	Descriptive	SCI (7dpi)+Vehicle (*n* = 9 zebrafish): 0.52 ± 0.04<br>SCI (7dpi)+ChABC (*n* = 8 zebrafish): 0.66 ± 0.06			
	Unpaired *t*-test	t = 0.744, df = 17 (Two-tailed)	SCI (14dpi)+Vehicle<br>SCI (14dpi)+ChABC	ns	*P* = 0.4665
	Descriptive	SCI (14dpi)+Vehicle (*n* = 8 zebrafish): 0.66 ± 0.05<br>SCI (14dpi)+ChABC (*n* = 11 zebrafish): 0.6 ± 0.05			
	Unpaired *t*-test	t = 2.69, df = 13 (Two-tailed)	SCI (21dpi)+Vehicle<br>SCI (21dpi)+ChABC	*	*P* = 0.0185
	Descriptive	SCI (21dpi)+Vehicle (*n* = 7 zebrafish): 0.81 ± 0.05<br>SCI (21dpi)+ChABC (*n* = 8 zebrafish): 0.61 ± 0.05			
**5G**	Unpaired *t*-test	t = 1.766, df = 15 (Two-tailed)	SCI (7dpi)+Vehicle<br>SCI (7dpi)+ChABC	ns	*P* = 0.0978
	Descriptive	SCI (7dpi)+Vehicle (*n* = 9 zebrafish): 0.5 ± 0.04<br>SCI (7dpi)+ChABC (*n* = 8 zebrafish): 0.64 ± 0.06			
	Unpaired *t*-test	t = 1.717, df = 17 (Two-tailed)	SCI (14dpi)+Vehicle<br>SCI (14dpi)+ChABC	ns	*P* = 0.1042
	Descriptive	SCI (14dpi)+Vehicle (*n* = 8 zebrafish): 0.69 ± 0.06<br>SCI (14dpi)+ChABC (*n* = 11 zebrafish): 0.57 ± 0.03			
	Unpaired *t*-test	t = 3.258, df = 13 (Two-tailed)	SCI (21dpi)+Vehicle<br>SCI (21dpi)+ChABC	**	*P* = 0.0062
	Descriptive	SCI (21dpi)+Vehicle (*n* = 7 zebrafish): 0.8 ± 0.05<br>SCI (21dpi)+ChABC (*n* = 8 zebrafish): 0.58 ± 0.04			
**5H (total distance)**	Unpaired *t*-test	t = 0.059, df = 64 (Two-tailed)	SCI (7dpi)+Vehicle<br>SCI (7dpi)+ChABC	ns	*P* = 0.9527
	Descriptive	SCI (7dpi)+Vehicle (*n* = 33 zebrafish): 224.9 ± 18.1<br>SCI (7dpi)+ChABC (*n* = 33 zebrafish): 227.3 ± 35.8			
	Unpaired *t*-test	t = 0.824, df = 37 (Two-tailed)	SCI (14dpi)+Vehicle<br>SCI (14dpi)+ChABC	ns	*P* = 0.4148
	Descriptive	SCI (14dpi)+Vehicle (*n* = 22 zebrafish): 369.1 ± 45.6<br>SCI (14dpi)+ChABC (*n* = 17 zebrafish): 428 ± 55.8			
	Unpaired *t*-test	t = 2.089, df = 17 (Two-tailed)	SCI (21dpi)+Vehicle<br>SCI (21dpi)+ChABC	ns	*P* = 0.052
	Descriptive	SCI (21dpi)+Vehicle (*n* = 8 zebrafish): 617.6 ± 168.5<br>SCI (21dpi)+ChABC (*n* = 11 zebrafish): 308.1 ± 35.3			
**5H (immobility index)**	Unpaired *t*-test	t = 0.059, df = 64 (Two-tailed)	SCI (7dpi)+Vehicle<br>SCI (7dpi)+ChABC	ns	*P* = 0.9531
	Descriptive	SCI (7dpi)+Vehicle (*n* = 33 zebrafish): 0.89 ± 0.01<br>SCI (7dpi)+ChABC (*n* = 33 zebrafish): 0.89 ± 0.02			
	Unpaired *t*-test	t = 0.369, df = 37 (Two-tailed)	SCI (14dpi)+Vehicle<br>SCI (14dpi)+ChABC	ns	*P* = 0.7136
	Descriptive	SCI (14dpi)+Vehicle (*n* = 22 zebrafish): 0.8 ± 0.02<br>SCI (14dpi)+ChABC (*n* = 17 zebrafish): 0.79 ± 0.03			

	Unpaired<br>*t*-test	t = 2.282, df = 17 (Two-tailed)		SCI (21dpi)+Vehicle<br>SCI (21dpi)+ChABC	*	$P = 0.0357$
	Descriptive	SCI (21dpi)+Vehicle (*n* = 8 zebrafish): 0.66 ± 0.09<br>SCI (21dpi)+ChABC (*n* = 11 zebrafish): 0.84 ± 0.02				
**5I**	Unpaired<br>*t*-test	t = 1.434, df = 53 (Two-tailed)		SCI (7dpi)+Vehicle<br>SCI (7dpi)+ChABC	ns	$P = 0.1576$
	Descriptive	SCI (7dpi)+Vehicle (*n* = 27 zebrafish): 229.4 ± 16.2<br>SCI (7dpi)+ChABC (*n* = 28 zebrafish): 268.9 ± 21.9				
	Unpaired<br>*t*-test	t = 0.826, df = 35 (Two-tailed)		SCI (14dpi)+Vehicle<br>SCI (14dpi)+ChABC	ns	$P = 0.414$
	Descriptive	SCI (14dpi)+Vehicle (*n* = 18 zebrafish): 339.88 ± 33.2<br>SCI (14dpi)+ChABC (*n* = 19 zebrafish): 375.9 ± 28.5				
	Unpaired<br>*t*-test	t = 0.252, df = 35 (Two-tailed)		SCI (21dpi)+Vehicle<br>SCI (21dpi)+ChABC	ns	$P = 0.8023$
	Descriptive	SCI (21dpi)+Vehicle (*n* = 17 zebrafish): 379.1 ± 27.4<br>SCI (21dpi)+ChABC (*n* = 20 zebrafish): 389.1 ± 28				
**SUPPLEMENTARY FIGURES**						
**S2A<br>(RMP)**	One-way ANOVA	$F_{(3, 76)} = 13.33$<br>$P < 0.0001$	Dunnett's test	Control<br>SCI (3dpi)	****	$P_{adj} < 0.0001$
				Control<br>SCI (7dpi)	*	$P_{adj} = 0.0119$
				Control<br>SCI (14dpi)	ns	$P_{adj} = 0.9746$
	Descriptive	Control (*n* = 40 neurons): -64.45 ± 0.6<br>SCI (3dpi) (*n* = 15 neurons): -56.98 ± 0.8<br>SCI (7dpi) (*n* = 10 neurons): -59.86 ± 2.1<br>SCI (14dpi) (*n* = 15 neurons): -64.93 ± 1.1				
**S2A<br>(Rinput)**	One-way ANOVA	$F_{(3, 76)} = 17.1$<br>$P < 0.0001$	Dunnett's test	Control<br>SCI (3dpi)	****	$P_{adj} < 0.0001$
				Control<br>SCI (7dpi)	****	$P_{adj} < 0.0001$
				Control<br>SCI (14dpi)	ns	$P_{adj} = 0.9911$
	Descriptive	Control (*n* = 40 neurons): 0.04 ± 0.001<br>SCI (3dpi) (*n* = 15 neurons): 0.06 ± 0.004<br>SCI (7dpi) (*n* = 10 neurons): 0.06 ± 0.006<br>SCI (14dpi) (*n* = 15 neurons): 0.04 ± 0.003				
**S2A<br>(threshold)**	One-way ANOVA	$F_{(3, 76)} = 1.268$<br>$P = 0.2915$	Dunnett's test	Control<br>SCI (3dpi)	ns	$P_{adj} = 0.6503$
				Control<br>SCI (7dpi)	ns	$P_{adj} = 0.5286$
				Control<br>SCI (14dpi)	ns	$P_{adj} = 0.2404$
	Descriptive	Control (*n* = 40 neurons): 9.21 ± 2.7<br>SCI (3dpi) (*n* = 15 neurons): 3.6 ± 4.4<br>SCI (7dpi) (*n* = 10 neurons): 1.52 ± 7.4<br>SCI (14dpi) (*n* = 15 neurons): -0.11 ± 4.4				
**S2A<br>(peak)**	One-way ANOVA	$F_{(3, 76)} = 0.332$<br>$P = 0.8017$	Dunnett's test	Control<br>SCI (3dpi)	ns	$P_{adj} = 0.8702$
				Control<br>SCI (7dpi)	ns	$P_{adj} = 0.7763$
				Control<br>SCI (14dpi)	ns	$P_{adj} = 0.9247$
	Descriptive	Control (*n* = 40 neurons): 35.69 ± 2.4<br>SCI (3dpi) (*n* = 15 neurons): 32.69 ± 2.4<br>SCI (7dpi) (*n* = 10 neurons): 31.3 ± 6.5<br>SCI (14dpi) (*n* = 15 neurons): 33.25 ± 3.0				
**S2A<br>(amplitude)**	One-way ANOVA	$F_{(3, 76)} = 1.81$<br>$P = 0.1525$	Dunnett's test	Control<br>SCI (3dpi)	ns	$P_{adj} = 0.1955$
				Control<br>SCI (7dpi)	ns	$P_{adj} = 0.6598$
				Control<br>SCI (14dpi)	ns	$P_{adj} = 0.1813$
	Descriptive	Control (*n* = 40 neurons): 26.94 ± 1.0<br>SCI (3dpi) (*n* = 15 neurons): 31.29 ± 2.4<br>SCI (7dpi) (*n* = 10 neurons): 29.77 ± 3.0<br>SCI (14dpi) (*n* = 15 neurons): 31.38 ± 2.0				
**S2A<br>(rheobase)**	One-way ANOVA	$F_{(3, 76)} = 12.71$<br>$P < 0.0001$	Dunnett's test	Control<br>SCI (3dpi)	****	$P_{adj} < 0.0001$
				Control<br>SCI (7dpi)	***	$P_{adj} = 0.0007$

				Control<br>SCI (14dpi)	ns	P_{adj} = 0.9995
	Descriptive	Control (*n* = 40 neurons): 1607 ± 67.1<br>SCI (3dpi) (*n* = 15 neurons): 1042 ± 58.4<br>SCI (7dpi) (*n* = 10 neurons): 1106 ± 121.0<br>SCI (14dpi) (*n* = 15 neurons): 1618 ± 70.7				
**S2A (half-width)**	One-way ANOVA	$F_{(3, 76)}$ = 3.308<br>*P* = 0.0246	Dunnett's test	Control<br>SCI (3dpi)	ns	P_{adj} = 0.0786
				Control<br>SCI (7dpi)	*	P_{adj} = 0.0266
				Control<br>SCI (14dpi)	ns	P_{adj} = 0.6844
	Descriptive	Control (*n* = 40 neurons): 0.3 ± 0.009<br>SCI (3dpi) (*n* = 15 neurons): 0.35 ± 0.02<br>SCI (7dpi) (*n* = 10 neurons): 0.37 ± 0.02<br>SCI (14dpi) (*n* = 15 neurons): 0.32 ± 0.01				
**S2A (AHP)**	One-way ANOVA	$F_{(3, 76)}$ = 1.366<br>*P* = 0.2597	Dunnett's test	Control<br>SCI (3dpi)	ns	P_{adj} = 0.199
				Control<br>SCI (7dpi)	ns	P_{adj} = 0.4699
				Control<br>SCI (14dpi)	ns	P_{adj} = 0.8056
	Descriptive	Control (*n* = 40 neurons): 8.88 ± 0.4<br>SCI (3dpi) (*n* = 15 neurons): 10.21 ± 0.5<br>SCI (7dpi) (*n* = 10 neurons): 9.99 ± 0.7<br>SCI (14dpi) (*n* = 15 neurons): 9.46 ± 0.6				
**S2B (RMP)**	One-way ANOVA	$F_{(3, 56)}$ = 25.84<br>*P* < 0.0001	Dunnett's test	Control<br>SCI (3dpi)	****	P_{adj} <0.0001
				Control<br>SCI (7dpi)	ns	P_{adj} = 0.0685
				Control<br>SCI (14dpi)	ns	P_{adj} = 0.5732
	Descriptive	Control (*n* = 20 neurons): -64.38 ± 0.5<br>SCI (3dpi) (*n* = 14 neurons): -53.9 ± 1.4<br>SCI (7dpi) (*n* = 16 neurons): -68.0 ± 1.3<br>SCI (14dpi) (*n* = 10 neurons): -66.4 ± 1.8				
**S2B (Rinput)**	One-way ANOVA	$F_{(3, 56)}$ = 19.51<br>*P* < 0.0001	Dunnett's test	Control<br>SCI (3dpi)	****	P_{adj} <0.0001
				Control<br>SCI (7dpi)	**	P_{adj} = 0.0023
				Control<br>SCI (14dpi)	ns	P_{adj} = 0.7167
	Descriptive	Control (*n* = 20 neurons): 101.8 ± 11.5<br>SCI (3dpi) (*n* = 14 neurons): 276.3 ± 24.26<br>SCI (7dpi) (*n* = 16 neurons): 183.3 ± 19.49<br>SCI (14dpi) (*n* = 10 neurons): 125.6 ± 12.0				
**S2B (threshold)**	One-way ANOVA	$F_{(3, 56)}$ = 8.534<br>*P* < 0.0001	Dunnett's test	Control<br>SCI (3dpi)	****	P_{adj} <0.0001
				Control<br>SCI (7dpi)	**	P_{adj} = 0.0083
				Control<br>SCI (14dpi)	ns	P_{adj} = 0.9265
	Descriptive	Control (*n* = 20 neurons): -20.03 ± 1.2<br>SCI (3dpi) (*n* = 14 neurons): -29.1 ± 1.5<br>SCI (7dpi) (*n* = 16 neurons): -25.94 ± 1.1<br>SCI (14dpi) (*n* = 10 neurons): -21.16 ± 2.2				
**S2B (peak)**	One-way ANOVA	$F_{(3, 56)}$ = 1.482<br>*P* = 0.2293	Dunnett's test	Control<br>SCI (3dpi)	ns	P_{adj} = 0.9858
				Control<br>SCI (7dpi)	ns	P_{adj} = 0.7128
				Control<br>SCI (14dpi)	ns	P_{adj} = 0.3941
	Descriptive	Control (*n* = 20 neurons): 1.199 ± 1.4<br>SCI (3dpi) (*n* = 14 neurons): 1.896 ± 2.1<br>SCI (7dpi) (*n* = 16 neurons): -0.924 ± 1.8<br>SCI (14dpi) (*n* = 10 neurons): 4.95 ± 1.6				
**S2B (amplitude)**	One-way ANOVA	$F_{(3, 56)}$ = 2.024<br>*P* = 0.121	Dunnett's test	Control<br>SCI (3dpi)	ns	P_{adj} = 0.9981
				Control<br>SCI (7dpi)	ns	P_{adj} = 0.1327
				Control<br>SCI (14dpi)	ns	P_{adj} = 0.2455

	Descriptive	Control (n = 20 neurons): 21.22 ± 0.7<br>SCI (3dpi) (n = 14 neurons): 21.48 ± 1.5<br>SCI (7dpi) (n = 16 neurons): 24.57 ± 1.3<br>SCI (14dpi) (n = 10 neurons): 24.47 ± 2.0				
**S2B (rheobase)**	One-way ANOVA	$F_{(3, 56)} = 6.788$<br>$P = 0.0006$	Dunnett's test	Control<br>SCI (3dpi)	***	$P_{adj} = 0.0005$
				Control<br>SCI (7dpi)	**	$P_{adj} = 0.0039$
				Control<br>SCI (14dpi)	ns	$P_{adj} = 0.5957$
	Descriptive	Control (n = 20 neurons): 269.0 ± 31.4<br>SCI (3dpi) (n = 14 neurons): 123.6 ± 21.3<br>SCI (7dpi) (n = 16 neurons): 150.9 ± 17.39<br>SCI (14dpi) (n = 10 neurons): 225.5 ± 29.4				
**S2B (half-width)**	One-way ANOVA	$F_{(3, 56)} = 2.946$<br>$P = 0.004$	Dunnett's test	Control<br>SCI (3dpi)	ns	$P_{adj} = 0.965$
				Control<br>SCI (7dpi)	ns	$P_{adj} = 0.1179$
				Control<br>SCI (14dpi)	ns	$P_{adj} = 0.6579$
	Descriptive	Control (n = 20 neurons): 0.41 ± 0.01<br>SCI (3dpi) (n = 14 neurons): 0.4 ± 0.008<br>SCI (7dpi) (n = 16 neurons): 0.45 ± 0.02<br>SCI (14dpi) (n = 10 neurons): 0.39 ± 0.01				
**S2B (AHP)**	One-way ANOVA	$F_{(3, 56)} = 1.947$<br>$P = 0.1325$	Dunnett's test	Control<br>SCI (3dpi)	ns	$P_{adj} = 0.9398$
				Control<br>SCI (7dpi)	ns	$P_{adj} = 0.7866$
				Control<br>SCI (14dpi)	ns	$P_{adj} = 0.1386$
	Descriptive	Control (n = 20 neurons): 2.24 ± 0.3<br>SCI (3dpi) (n = 14 neurons): 1.96 ± 0.4<br>SCI (7dpi) (n = 16 neurons): 2.69 ± 0.4<br>SCI (14dpi) (n = 10 neurons): 3.54 ± 0.6				
**S3A**	Simple linear regression	**pMNs**<br>Equation: $Y = 19.92*X + 200.3$<br>R^2: 0.00706<br>**dV2a-INs**<br>Equation: $Y = 13.62*X + 71.8$<br>R^2: 0.00856				
**S4A**	One-way ANOVA	$F_{(3, 54)} = 43.69$<br>$P < 0.0001$	Tukey's test	Control<br>SCI (3dpi)	****	$P_{adj} < 0.0001$
				Control<br>SCI (3dpi)+ChABC (4U)	ns	$P_{adj} = 0.3753$
				Control<br>SCI (3dpi)+ChABC (8U)	****	$P_{adj} < 0.0001$
				SCI (3dpi)<br>SCI (3dpi)+ChABC (4U)	****	$P_{adj} < 0.0001$
				SCI (3dpi)<br>SCI (3dpi)+ChABC (8U)	****	$P_{adj} < 0.0001$
				SCI (3dpi)+ChABC (4U)<br>SCI (3dpi)+ChABC (8U)	***	$P_{adj} = 0.0002$
	Descriptive	Control (n = 24 zebrafish): 0.51 ± 0.02<br>SCI (3dpi) (n = 15 zebrafish): 0.78 ± 0.03<br>SCI (3dpi)+ChABC (4U) (n = 11 zebrafish): 0.43 ± 0.04<br>SCI (3dpi)+ChABC (8U) (n = 8 zebrafish): 0.17 ± 0.03				
**S5A (Adaptation index)**	Unpaired t-test	t = 0.34, df = 19 (Two-tailed)		SCI (3dpi)<br>SCI (3dpi)+Vehicle	ns	$P = 0.7324$
	Descriptive	SCI (3dpi) (n = 15 neurons): 0.22 ± 0.03<br>SCI (3dpi)+Vehicle (n = 6 neurons): 0.2 ± 0.04				
**S5A (RMP)**	Unpaired t-test	t = 0.43, df = 19 (Two-tailed)		SCI (3dpi)<br>SCI (3dpi)+Vehicle	ns	$P = 0.6662$
	Descriptive	SCI (3dpi) (n = 15 neurons): -56.98 ± 0.86<br>SCI (3dpi)+Vehicle (n = 6 neurons): -56.2 ± 1.84				
**S5A (Rinput)**	Unpaired t-test	t = 0.76, df = 19 (Two-tailed)		SCI (3dpi)<br>SCI (3dpi)+Vehicle	ns	$P = 0.451$
	Descriptive	SCI (3dpi) (n = 15 neurons): 0.06 ± 0.004<br>SCI (3dpi)+Vehicle (n = 6 neurons): 0.07 ± 0.007				
**S5A (Rheobase)**	Unpaired t-test	t = 0.06, df = 19 (Two-tailed)		SCI (3dpi)<br>SCI (3dpi)+Vehicle	ns	$P = 0.949$
	Descriptive	SCI (3dpi) (n = 15 neurons): 1042 ± 58.49<br>SCI (3dpi)+Vehicle (n = 6 neurons): 1049 ± 73.48				

**S5A (AP amplitude)**	Unpaired *t*-test	t = 0.47, df = 19 (Two-tailed)		SCI (3dpi)<br>SCI (3dpi)+Vehicle	ns	*P* = 0.6436
	Descriptive	SCI (3dpi) (*n* = 15 neurons): 31.29 ± 2.45<br>SCI (3dpi)+Vehicle (*n* = 6 neurons): 29.2 ± 3.39				
**S5A (AHP amplitude)**	Unpaired *t*-test	t = 0.15, df = 19 (Two-tailed)		SCI (3dpi)<br>SCI (3dpi)+Vehicle	ns	*P* = 0.8769
	Descriptive	SCI (3dpi) (*n* = 15 neurons): 10.21 ± 0.51<br>SCI (3dpi)+Vehicle (*n* = 6 neurons): 10.36 ± 0.64				
**S5A (Half-width)**	Unpaired *t*-test	t = 1.45, df = 19 (Two-tailed)		SCI (3dpi)<br>SCI (3dpi)+Vehicle	ns	*P* = 0.1631
	Descriptive	SCI (3dpi) (*n* = 15 neurons): 0.35 ± 0.02<br>SCI (3dpi)+Vehicle (*n* = 6 neurons): 0.3 ± 0.01				
**S5A (Threshold)**	Unpaired *t*-test	t = 0.72, df = 19 (Two-tailed)		SCI (3dpi)<br>SCI (3dpi)+Vehicle	ns	*P* = 0.4744
	Descriptive	SCI (3dpi) (*n* = 15 neurons): 3.6 ± 4.4<br>SCI (3dpi)+Vehicle (*n* = 6 neurons): -2.11 ± 5.2				
**S5A (EPSP freq)**	Unpaired *t*-test	t = 0.12, df = 9 (Two-tailed)		SCI (3dpi)<br>SCI (3dpi)+Vehicle	ns	*P* = 0.9002
	Descriptive	SCI (3dpi) (*n* = 7 neurons): 34.67 ± 6.17<br>SCI (3dpi)+vehicle (*n* = 4 neurons): 33.45 ± 6.02				
**S5A (EPSP amplitude)**	Unpaired *t*-test	t = 0.12, df = 9 (Two-tailed)		SCI (3dpi)<br>SCI (3dpi)+Vehicle	ns	*P* = 0.9067
	Descriptive	SCI (3dpi) (*n* = 7 neurons): 0.39 ± 0.05<br>SCI (3dpi)+Vehicle (*n* = 4 neurons): 0.4 ± 0.05				
**S5B (Adaptation index)**	Unpaired *t*-test	t = 0.77, df = 18 (Two-tailed)		SCI (3dpi)<br>SCI (3dpi)+Vehicle	ns	*P* = 0.4466
	Descriptive	SCI (3dpi) (*n* = 14 neurons): 0.62 ± 0.04<br>SCI (3dpi)+Vehicle (*n* = 6 neurons): 0.69 ± 0.07				
**S5B (RMP)**	Unpaired *t*-test	t = 2.09, df = 18 (Two-tailed)		SCI (3dpi)<br>SCI (3dpi)+Vehicle	ns	*P* = 0.0510
	Descriptive	SCI (3dpi) (*n* = 14 neurons): -53.9 ± 1.47<br>SCI (3dpi)+Vehicle (*n* = 6 neurons): -59.28 ± 1.89				
**S5B (*R*input)**	Unpaired *t*-test	t = 0.63, df = 18 (Two-tailed)		SCI (3dpi)<br>SCI (3dpi)+Vehicle	ns	*P* = 0.5307
	Descriptive	SCI (3dpi) (*n* = 14 neurons): 276.3 ± 24.26<br>SCI (3dpi)+Vehicle (*n* = 6 neurons): 305.7 ± 42.09				
**S5B (Rheobase)**	Unpaired *t*-test	t = 0.87, df = 18 (Two-tailed)		SCI (3dpi)<br>SCI (3dpi)+Vehicle	ns	*P* = 0.3947
	Descriptive	SCI (3dpi) (*n* = 14 neurons): 123.6 ± 21.33<br>SCI (3dpi)+Vehicle (*n* = 6 neurons): 156.7 ± 29.51				
**S5B (AP amplitude)**	Unpaired *t*-test	t = 1.32, df = 18 (Two-tailed)		SCI (3dpi)<br>SCI (3dpi)+Vehicle	ns	*P* = 0.2003
	Descriptive	SCI (3dpi) (*n* = 14 neurons): 21.4 ± 1.53<br>SCI (3dpi)+Vehicle (*n* = 6 neurons): 26.3 ± 4.37				
**S5B (AHP amplitude)**	Unpaired *t*-test	t = 1.91, df = 18 (Two-tailed)		SCI (3dpi)<br>SCI (3dpi)+Vehicle	ns	*P* = 0.0720
	Descriptive	SCI (3dpi) (*n* = 14 neurons): 1.96 ± 0.4<br>SCI (3dpi)+Vehicle (*n* = 6 neurons): 3.7 ± 0.8				
**S5B (Half-width)**	Unpaired *t*-test	t = 0.9, df = 18 (Two-tailed)		SCI (3dpi)<br>SCI (3dpi)+Vehicle	ns	*P* = 0.3791
	Descriptive	SCI (3dpi) (*n* = 14 neurons): 0.4 ± 0.008<br>SCI (3dpi)+Vehicle (*n* = 6 neurons): 0.42 ± 0.009				
**S5B (Threshold)**	Unpaired *t*-test	t = 0.81, df = 18 (Two-tailed)		SCI (3dpi)<br>SCI (3dpi)+Vehicle	ns	*P* = 0.4250
	Descriptive	SCI (3dpi) (*n* = 14 neurons): -29.1 ± 1.5<br>SCI (3dpi)+Vehicle (*n* = 6 neurons): -26.7 ± 2.7				
**S5B (EPSP freq)**	Unpaired *t*-test	t = 0.36, df = 11 (Two-tailed)		SCI (3dpi)<br>SCI (3dpi)+Vehicle	ns	*P* = 0.7196
	Descriptive	SCI (3dpi) (*n* = 9 neurons): 19.7 ± 0.9<br>SCI (3dpi)+Vehicle (*n* = 4 neurons): 19.1 ± 1.4				
**S5B (EPSP amplitude)**	Unpaired *t*-test	t = 0.07, df = 11 (Two-tailed)		SCI (3dpi)<br>SCI (3dpi)+Vehicle	ns	*P* = 0.9382
	Descriptive	SCI (3dpi) (*n* = 9 neurons): 1.35 ± 0.17<br>SCI (3dpi)+Vehicle (*n* = 4 neurons): 1.32 ± 0.2				
**S6B (total distance)**	One-way ANOVA	$F_{(4, 77)} = 7.409$<br>$P < 0.0001$	Dunnett's test	Control<br>SCI (3dpi)	**	$P_{adj} = 0.0032$
				Control<br>SCI (7dpi)	**	$P_{adj} = 0.0035$
				Control<br>SCI (14dpi)	ns	$P_{adj} = 0.1612$
				Control<br>SCI (21dpi)	ns	$P_{adj} = 0.9760$

	Descriptive	Control (*n* = 7 zebrafish): 567.5 ± 192.6<br>SCI (3dpi) (*n* = 12 zebrafish): 172.5 ± 22.5<br>SCI (7dpi) (*n* = 33 zebrafish): 224.9 ± 18.1<br>SCI (14dpi) (*n* = 22 zebrafish): 369.1 ± 45.6<br>SCI (21dpi) (*n* = 8 zebrafish): 615.8 ± 168.5				
**S6B (turns)**	One-way ANOVA	$F_{(4, 77)} = 8.826$<br>$P < 0.0001$	Dunnett's test	Control<br>SCI (3dpi)	***	$P_{adj} = 0.0004$
				Control<br>SCI (7dpi)	***	$P_{adj} = 0.0003$
				Control<br>SCI (14dpi)	**	$P_{adj} = 0.0084$
				Control<br>SCI (21dpi)	ns	$P_{adj} > 0.9999$
	Descriptive	Control (*n* = 7 zebrafish): 40.86 ± 14.5<br>SCI (3dpi) (*n* = 12 zebrafish): 4.0 ± 1.1<br>SCI (7dpi) (*n* = 33 zebrafish): 7.84 ± 1.2<br>SCI (14dpi) (*n* = 22 zebrafish): 15.0 ± 2.7<br>SCI (21dpi) (*n* = 8 zebrafish): 40.38 ± 15.9				
**S6B (immobility)**	One-way ANOVA	$F_{(4, 77)} = 7.287$<br>$P < 0.0001$	Dunnett's test	Control<br>SCI (3dpi)	*	$P_{adj} = 0.0286$
				Control<br>SCI (7dpi)	*	$P_{adj} = 0.03$
				Control<br>SCI (14dpi)	ns	$P_{adj} = 0.6337$
				Control<br>SCI (21dpi)	ns	$P_{adj} = 0.4262$
	Descriptive	Control (*n* = 7 zebrafish): 0.75 ± 0.07<br>SCI (3dpi) (*n* = 12 zebrafish): 0.91 ± 0.01<br>SCI (7dpi) (*n* = 33 zebrafish): 0.89 ± 0.01<br>SCI (14dpi) (*n* = 22 zebrafish): 0.8 ± 0.02<br>SCI (21dpi) (*n* = 8 zebrafish): 0.66 ± 0.09				
**S6B (mean acceleration)**	One-way ANOVA	$F_{(4, 77)} = 1.201$<br>$P = 0.3174$	Dunnett's test	Control<br>SCI (3dpi)	ns	$P_{adj} = 0.4343$
				Control<br>SCI (7dpi)	ns	$P_{adj} = 0.6971$
				Control<br>SCI (14dpi)	ns	$P_{adj} = 0.158$
				Control<br>SCI (21dpi)	ns	$P_{adj} = 0.8265$
	Descriptive	Control (*n* = 7 zebrafish): 0.007 ± 0.005<br>SCI (3dpi) (*n* = 12 zebrafish): 0.001 ± 0.001<br>SCI (7dpi) (*n* = 33 zebrafish): 0.003 ± 0.001<br>SCI (14dpi) (*n* = 22 zebrafish): -0.0002 ± 0.002<br>SCI (21dpi) (*n* = 8 zebrafish): 0.003 ± 0.002				
**S6B (activity)**	One-way ANOVA	$F_{(4, 77)} = 8.402$<br>$P < 0.0001$	Dunnett's test	Control<br>SCI (3dpi)	**	$P_{adj} = 0.0046$
				Control<br>SCI (7dpi)	**	$P_{adj} = 0.0077$
				Control<br>SCI (14dpi)	ns	$P_{adj} = 0.1485$
				Control<br>SCI (21dpi)	ns	$P_{adj} = 0.5799$
	Descriptive	Control (*n* = 7 zebrafish): 0.04 ± 0.01<br>SCI (3dpi) (*n* = 12 zebrafish): 0.005 ± 0.001<br>SCI (7dpi) (*n* = 33 zebrafish): 0.011 ± 0.001<br>SCI (14dpi) (*n* = 22 zebrafish): 0.022 ± 0.003<br>SCI (21dpi) (*n* = 8 zebrafish): 0.053 ± 0.01				
**S6C (total distance)**	One-way ANOVA	$F_{(4, 69)} = 4.814$<br>$P = 0.0017$	Dunnett's test	Control<br>SCI (3dpi)+ChABC	**	$P_{adj} = 0.007$
				Control<br>SCI (7dpi)+ChABC	**	$P_{adj} = 0.0034$
				Control<br>SCI (14dpi)+ChABC	ns	$P_{adj} = 0.4461$
				Control<br>SCI (21dpi)+ChABC	ns	$P_{adj} = 0.0805$
	Descriptive	Control (*n* = 7 zebrafish): 567.5 ± 192.6<br>SCI (3dpi)+ChABC (*n* = 6 zebrafish): 144.5 ± 22.5<br>SCI (7dpi)+ChABC (*n* = 33 zebrafish): 227.3 ± 35.8<br>SCI (14dpi)+ChABC (*n* = 17 zebrafish): 428.0 ± 55.8<br>SCI (21dpi)+ChABC (*n* = 11 zebrafish): 309.4 ± 35.0				
**S6C (turns)**	One-way ANOVA	$F_{(4, 69)} = 6.47$<br>$P = 0.0002$	Dunnett's test	Control<br>SCI (3dpi)+ChABC	**	$P_{adj} = 0.0017$

				Control<br>SCI (7dpi)+ChABC	****	P_{adj} <0.0001
				Control<br>SCI (14dpi)+ChABC	ns	P_{adj} = 0.0546
				Control<br>SCI (21dpi)+ChABC	**	P_{adj} = 0.0077
	Descriptive	Control (*n* = 7 zebrafish): 40.86 ± 14.5<br>SCI (3dpi)+ChABC (*n* = 6 zebrafish): 5.5 ± 1.6<br>SCI (7dpi)+ChABC (*n* = 33 zebrafish): 7.78 ± 2.6<br>SCI (14dpi)+ChABC (*n* = 17 zebrafish): 22.06 ± 3.5<br>SCI (21dpi)+ChABC (*n* = 11 zebrafish): 14.36 ± 3.2				
**S6C (immobility)**	One-way ANOVA	$F_{(4, 69)} = 3.845$<br>$P = 0.007$	Dunnett's test	Control<br>SCI (3dpi)+ChABC	*	P_{adj} = 0.0211
				Control<br>SCI (7dpi)+ChABC	*	P_{adj} = 0.0268
				Control<br>SCI (14dpi)+ChABC	ns	P_{adj} = 0.8561
				Control<br>SCI (21dpi)+ChABC	ns	P_{adj} = 0.2645
	Descriptive	Control (*n* = 7 zebrafish): 0.75 ± 0.07<br>SCI (3dpi)+ChABC (*n* = 6 zebrafish): 0.94 ± 0.01<br>SCI (7dpi)+ChABC (*n* = 33 zebrafish): 0.89 ± 0.02<br>SCI (14dpi)+ChABC (*n* = 17 zebrafish): 0.79 ± 0.03<br>SCI (21dpi)+ChABC (*n* = 11 zebrafish): 0.84 ± 0.02				
**S6C (mean acceleration)**	One-way ANOVA	$F_{(4, 69)} = 0.948$<br>$P = 0.4414$	Dunnett's test	Control<br>SCI (3dpi)+ChABC	ns	P_{adj} = 0.8866
				Control<br>SCI (7dpi)+ChABC	ns	P_{adj} = 0.7936
				Control<br>SCI (14dpi)+ChABC	ns	P_{adj} = 0.2247
				Control<br>SCI (21dpi)+ChABC	ns	P_{adj} = 0.8935
	Descriptive	Control (*n* = 7 zebrafish): 0.007 ± 0.005<br>SCI (3dpi)+ChABC (*n* = 6 zebrafish): 0.0005 ± 0.0002<br>SCI (7dpi)+ChABC (*n* = 33 zebrafish): 0.001 ± 0.001<br>SCI (14dpi)+ChABC (*n* = 17 zebrafish): -0.007 ± 0.007<br>SCI (21dpi)+ChABC (*n* = 11 zebrafish): 0.001 ± 0.0001				
**S6C (activity)**	One-way ANOVA	$F_{(4, 69)} = 3.777$<br>$P = 0.007$	Dunnett's test	Control<br>SCI (3dpi)+ChABC	*	P_{adj} = 0.0143
				Control<br>SCI (7dpi)+ChABC	**	P_{adj} = 0.0076
				Control<br>SCI (14dpi)+ChABC	ns	P_{adj} = 0.3706
				Control<br>SCI (21dpi)+ChABC	ns	P_{adj} = 0.206
	Descriptive	Control (*n* = 7 zebrafish): 0.04 ± 0.01<br>SCI (3dpi)+ChABC (*n* = 6 zebrafish): 0.005 ± 0.002<br>SCI (7dpi)+ChABC (*n* = 33 zebrafish): 0.01 ± 0.003<br>SCI (14dpi)+ChABC (*n* = 17 zebrafish): 0.02 ± 0.004<br>SCI (21dpi)+ChABC (*n* = 11 zebrafish): 0.02 ± 0.003				

Table S2: Antibodies and Streptavidins Used[1]

Antigen	Host	Source	Code	Dilution
Primary				
α-Tubulin	Mouse	Sigma-Aldrich	T5168; RRID:AB_477579	1:500
ChAT	Goat	Millipore	AB144P; RRID: AB_2079751	1:150
c-Fos	Rabbit	GeneTex	GTX129846; RRID:AB_2886108	1:200
GFP	Chicken	GeneTex	GTX129846; RRID:AB_2886108	1:200
SV2	Mouse	Abcam	Ab13970; RRID:AB_300798	1:500
Secondary				
Mouse IgG-647	Donkey	ThermoFisher	A-31571; RRID: AB_162542	1:500
Mouse IgG-568	Donkey	ThermoFisher	A-10037; RRID: AB_2534013	1:500
Mouse IgG-488	Donkey	ThermoFisher	A-21202; RRID: AB_141607	1:500
Rabbit IgG-488	Donkey	ThermoFisher	A-21206; RRID: AB_2535792	1:500
Rabbit IgG-568	Donkey	ThermoFisher	A-10042; RRID: AB_2534017	1:500
Chicken IgY-FITC	Donkey	ThermoFisher	SA1-72000; RRID: AB_923386	1:500
Mouse IgG-Biotinylated	Horse	VectorLaboratories	BA-2000; RRID: AB_2313581	1:200
Rabbit IgG-Biotinylated	Horse	VectorLaboratories	BA-1100; RRID: AB_2336201	1:200
Streptavidin				
Alexa Fluor 488		ThermoFisher	S32354; RRID: AB_2315383	1:500
Alexa Fluor 555		ThermoFisher	S32355; RRID: AB_2571525	1:500
Alexa Fluor 647		ThermoFisher	S32357; RRID: AB_2336066	1:500

[1]c-Fos; Fos proto-oncogene; ChAT, choline-acetyltransferase; GFP, green fluorescent protein; SV2, Synaptic vesicle glycoprotein 2A.
